# Effectiveness and cost-effectiveness of human papillomavirus vaccination strategies among men who have sex with men in China: a modeling study

**DOI:** 10.3389/fimmu.2023.1197191

**Published:** 2023-06-22

**Authors:** Yuwei Li, Yi-Fan Lin, Xinsheng Wu, Xinyi Zhou, Tian Tian, Zhihui Guo, Leiwen Fu, Luoyao Yang, Zhen Lu, Song Fan, Yong Lu, Wujian Ke, Huachun Zou

**Affiliations:** ^1^ School of Public Health (Shenzhen), Sun Yat-sen University, Shenzhen, China; ^2^ School of Public Health, Southwest Medical University, Sichuan, China; ^3^ College of Public Health and Health Professions, Guizhou Medical University, Guizhou, China; ^4^ Department of Sexually Transmitted Disease (STD) Clinic, Dermatology Hospital of Southern Medical University, Guangzhou, China; ^5^ Kirby Institute, University of New South Wales, Kensington, NSW, Australia

**Keywords:** human papillomavirus, vaccination strategy, men who have sex with men (MSM), Markov model, cost-effectiveness analyses

## Abstract

**Introduction:**

The health and economic benefits of human papillomavirus (HPV) vaccination targeted at men who have sex with men (MSM) in developing settings have been rarely assessed. This study aimed to evaluate the effectiveness and cost-effectiveness of different HPV vaccination strategies among MSM in China.

**Methods:**

A Markov model was developed to simulate HPV transmission dynamics among a total of 30.73 million MSM in China. The corresponding natural history included 6 states: susceptible, infected with low-risk subtypes, high-risk subtypes, anogenital warts and anal cancer, and deaths from anal cancer. MSM were divided into three age groups with cut-off points of 27 and 45 years. Alternative vaccination strategies were built by allocating bivalent, quadrivalent, nine-valent, or no vaccine to each of the groups. We generated the prevented infections and deaths by vaccination compared with baseline (no vaccination) and calculated incremental cost-effectiveness ratios (ICERs) to determine the optimal strategy.

**Results:**

The model showed that in 10 years, at baseline, the existing cases of anogenital warts would reach 5,464,225 (IQR, 4,685,708-6,174,175); that of anal cancer would reach 1,922.95 (1,716.56-2,119.93), resulting in 940.55 (732.27-1,141.87) deaths. Under 50% vaccination coverage among one age group, the prevented cases of anogenital warts were maximized with quadrivalent vaccines allocated to MSM aged 27-45 years; that of anal cancer were maximized when offering nine-valent vaccines to the same group. Under 50% vaccination coverage among all groups, the lowest ICER (34,098.09 USD/QALY, 31,146.54-37,062.88) was reached when only quadrivalent vaccines were provided. Based on this strategy, when the annual vaccination rate increased by 30%, the ICER (33,521.75 USD/QALY, 31,040.73-36,013.92) would fall below three times China’s per capita GDP. When the vaccine price decreased by 60%, the ICER was reduced to 7,344.44 USD/QALY (4,392.89-10,309.23), indicating good cost-effectiveness taking China’s per capita GDP as a threshold.

**Conclusions:**

HPV vaccination can effectively reduce the prevalence and mortality of related diseases among MSM in China, especially quadrivalent vaccines for anogenital warts and nine-valent vaccines for anal cancer. MSM aged 27-45 years were the optimal group for vaccination. Annual vaccination and appropriate adjustment of vaccine price are necessary to further improve the cost-effectiveness.

## 1 Introduction

Human papillomavirus (HPV) infection and related morbidities are a major threat to human health (1). HPV is generally divided into low-risk (HPV-6, 11 and etc) and high-risk (HPV-16, 18, 31, 33 and etc) subtypes (2). Low-risk HPV infection can cause anogenital warts (3), while the persistent infection of high-risk HPV can lead to abnormal neoplasia which ultimately manifests as various cancers (4, 5). Almost all HPV-associated cancers could be directly attributed to HPV according to the United States Cancer Statistics (6). As shown in a study in Australia, HPV was detected in 85% of rectal cancers, 70% of cervical cancers, 50% of penile cancers, and 40% of vulval cancers (7). In China, the age-standardized incidence and mortality rate of HPV-attributable cancers demonstrated upward trends, as is presented in a disease burden analysis (8).

Men who have sex with men (MSM) are at high risk of HPV transmission (9). A study in Italy found that, the prevalence of any-type HPV infection in MSM was 48.9% (10), and that for HIV-positive MSM reached 60.0%, among whom 63.3% suffered high-risk HPV infection (5). Besides, the lack of self-protection perception during sexual behaviors has made MSM a population with a high HPV incidence (9). As is shown in a cohort study in Australia, the incidence of high-risk anal HPV was up to 77/100 person-years for those even at a relatively young age (11). The clearance rate of HPV among MSM, which was only 75.5/1000 person-years for high-risk anal HPV infection concluded in a cohort study in Italy, is also far below that among heterosexual males (12). Studies have shown that MSM are at higher risk of HPV persistent infection and cancer development, especially anal cancer, the incidence of which is 20 times that of heterosexual men (13). In China, the huge population base of MSM exacerbates the burdens of HPV-related diseases, which has led to the necessity of targeted protective strategies.

Fortunately, HPV vaccines are efficacious in preventing the infection of several carcinogenic HPV subtypes, and have become the most effective intervention in containing HPV-related diseases (14). There are three categories of preventive HPV vaccines at current, including bivalent (GlaxoSmithKline and Vantage Biopharmaceutical), quadrivalent (Merck), and nine-valent (Merck) vaccines (15). Bivalent vaccines cover HPV subtypes 16 and 18. On this basis, quadrivalent vaccines additionally cover subtypes 6 and 11, providing continuous prevention against anogenital warts for at least 10 years with 90% efficacy, according to a long-term follow-up (16). Nine-valent vaccines extend the prevention effect to HPV-31, 33, 45, 52, and 58 subtypes. A modeling study has found that nine-valent vaccination at appropriate ages could avert over 400 thousand HPV-related cancers among males (17). HPV vaccination plans have been involved in the National Immunity Program (NIP) of 66.49% of World Health Organization (WHO) member states (18). However, even in Europe, with relatively developed economies, funded vaccination was implemented in only 47% of countries as of January 2020, suggesting an urgent demand for further economic evaluations of HPV vaccines (19).

MSM have been recommended HPV vaccination globally (20). In China, the phase III clinical trial on the indication of recombinant nine-valent HPV vaccine (Beijing Health Guard Biotechnology Inc.) among men has been launched, where 1200 homosexual men will be recruited as volunteers (21). In order to reinforce targeted protection for high-risk groups and promote herd immunity, it is crucial to explore the transmission dynamics of HPV and develop reasonable vaccination strategies among MSM. However, current HPV transmission models primarily focus on women or heterosexual men. Among the few studies targeted at MSM, Zhang et al. investigated the effectiveness and cost-effectiveness of HPV vaccination programs in Australia, and the cost-effectiveness of HPV vaccination was also estimated by Kim et al. in the USA (22, 23). The health and economic benefits of HPV vaccination among MSM were rarely assessed in developing settings. Therefore, this study aimed to simulate the transmission dynamics of HPV and evaluate the effectiveness and cost-effectiveness of different HPV vaccination strategies among MSM in China.

## 2 Materials and methods

### 2.1 Study design and data sources

We developed an age-stratified multi-state Markov model to simulate HPV transmission among MSM in China. A transition probability matrix with disease-related parameters was constructed for model calculation. MSM were divided into three age groups with cut-off points of 27 and 45 years. HPV vaccination was considered as a preventive intervention and was conducted cost-effectiveness evaluation. All data were collected from government websites and published literature. Details about all parameters applied in the model are presented in **Table 1**.

**Table 1:** Parameters used for the construction of the Markov transmission matrix and outcome calculation.

(a) Population size and/or proportion of different age groups					
	Group^a^	Symbol	Value	Range	Source
**Size**	Total	n	30,728,379	[30,677,941-30,778,818]	(24–26)
**Proportion**	Group1	n_1_/n	27.30%	[25%-29%]	(27–29)
	Group2	n_2_/n	68.60%	[65%-72%]	(27–29)
	Group3	n_3_/n	4.10%	[3%-6%]	(27–29)
(b) Incidence rate of HPV infection					
	Status	Symbol	Value	Range	Source
**Incidence rate** **(overall)**	L	p_L_	0.174	[0.119-0.238]	(30)
	H	p_H_	0.205	[0.148-0.277]	(30)
(c) Transmission rate of Markov matrix					
	Transmission	Symbol	Value	Range	Source
**Development**	L->G	p_LG_	0.29	[0.19-0.4]	(31)
	H->A	p_HA_	0.0000379	[0.000033-0.00004]	(32); calculated
**Recovery**	L->S	γ_L_	0.898	[0.815-0.988]	(33)
	H->S	γ_H_	0.752	[0.682-0.800]	(33)
	G->S	γ_G_	0.19	[0.102-0.212]	(34)
**Death**	A->D	d_A_	0.0688	[0.0408-0.1374]	(32); calculated
**Natural death**	Group1/2/3->D0	d_0_	0.00718	0.00718	(26)
**Natural birth**	->Group1	b_0_	0.00752	0.00752	(26)
**Aging**	Group1->Group2	μ_1_	1/17	assumed	assumed^b^
	Group2->Group3	μ_2_	1/19	assumed	assumed^b^
(d) Vaccination parameters					
	Category	Symbol	Value	Range	Source
**VE**	Bivalent	ω_2_	94.0%	[80.0%-99.0%]	(35, 36)
	Quadrivalent	ω_4_	90.4%	[69.2%-98.1%]	(37, 38)
	Nine-valent	ω_9_	96.7%	[80.9%-99.8%]	(39)
**Vaccinating rate**	/	β	1%	[1%-10%]	assumed^b^
**Coverage**	Bivalent	α_2_	50%	[30%-70%]	assumed^b^
	Quadrivalent	α_4_	50%	[30%-70%]	assumed^b^
	Nine-valent	α_9_	50%	[30%-70%]	assumed^b^
(e) Cost-effectiveness evaluation parameters					
	Category	Symbol	Value	Range	Source
**Cost**	Bivalent	c_2_	51.30	[50.72-57.97]	(36, 40, 41)
	Quadrivalent	c_4_	119.28	[115.94-130.43]	(41)
	Nine-valent	c_9_	191.74	[188.41-210.14]	(41)
	Treatment for anogenital warts^c^	c_g_	511.73	[266.67-1,008.10]	Dermatology Hospital of Southern Medical University
	Treatment for anal cancer^c^	c_a_	11,594.20	[10,869.57-14,492.75]	Sun Yat-sen University Cancer Center
	Discount rate	ω	0.03	[0.0-0.08]	(42)
**QALY**	S	q_S_	1	1	assumed
	L	q_L_	1	1	assumed
	H	q_H_	1	1	assumed
	G	q_G_	0.91	[0.88-0.93]	(22)
	A	q_A_	0.68	[0.65-0.70]	(22)
	D	q_D_	0	0	assumed

HPV, human papillomavirus; VE, vaccination efficacy; QALY, quality-adjusted life years.

a Group1: MSM aged <27 years; Group2: MSM aged 27-45 years; Group3: MSM aged >45 years.

b These parameters were conducted sensitivity analyses.

c Data on costs collected or consulted from institutions in China were adjusted to USD in 2022.

### 2.2 Model overview

#### 2.2.1 Model structure

**Figure 1:**
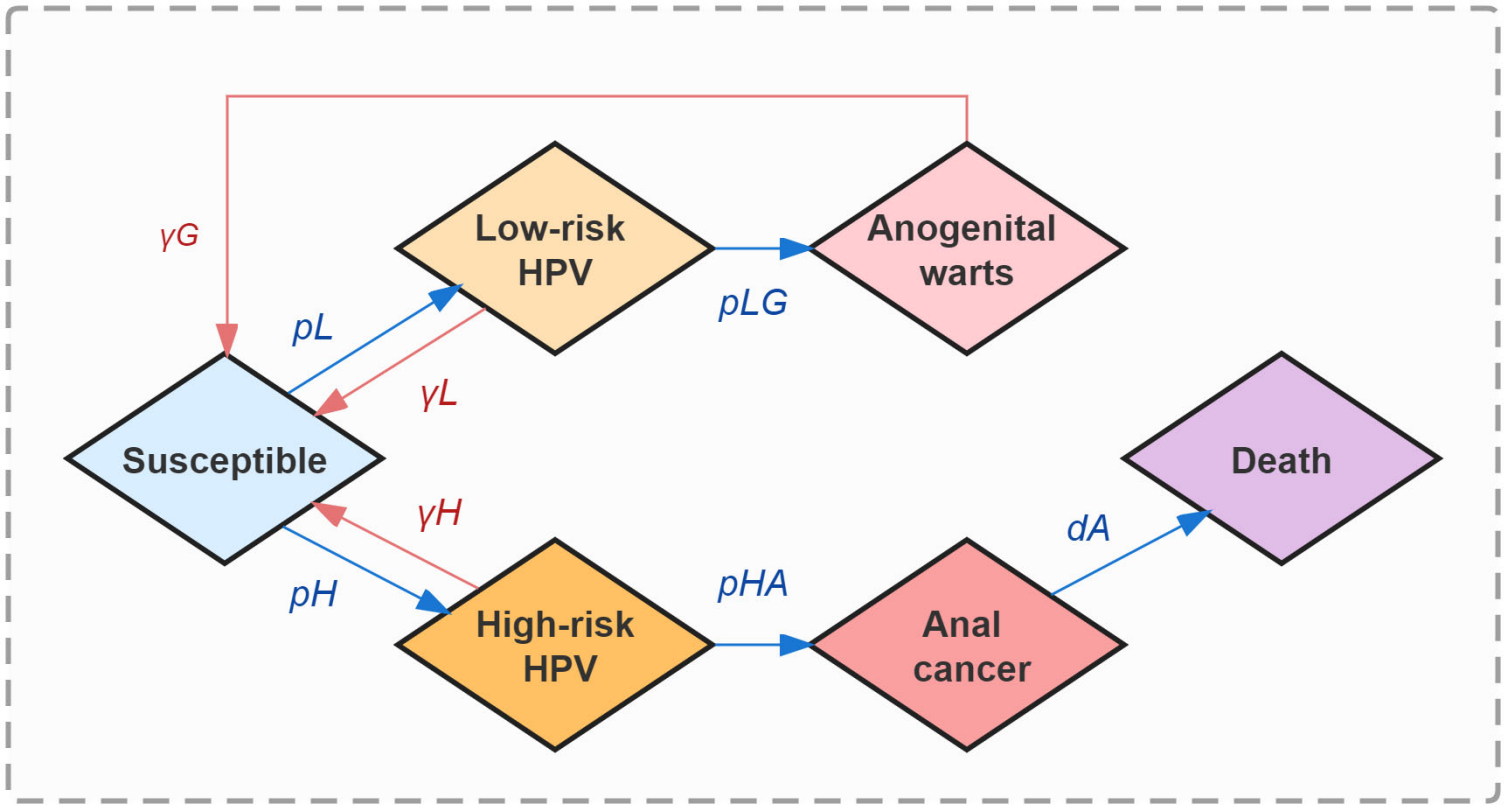
The natural history of HPV dynamic transmission among MSM.

The natural history of HPV infection, development, and clearance among MSM was represented in 6 states: susceptible (S, the initial state), infected with HPV low-risk subtypes (L), infected with HPV high-risk subtypes (H), anogenital warts (G), anal cancer (A), and deaths from anal cancer (D, the absorbing state). In the model simulation, each susceptible individual could be infected with either low-risk or high-risk HPV subtypes with different force transmission rates, and will hence enter the corresponding status L or H respectively. Individuals persistently infected with low-risk HPV have a chance for anogenital warts development, otherwise, they will shed low-risk HPV and re-enter the susceptible state (S). Those infected with anogenital warts will be likely to recover under proper treatment. Similarly, anal cancer could be triggered by persistent infection of high-risk HPV, except that we assumed individuals will not completely recover from formed cancerization. Once entering status A, an individual will be provided cancer therapy and face the risk of death. (**Figure 1**)

The total MSM population (n, 30.73 million) in China was initially included in the model. In order to better fit the real-world epidemic situation of HPV-related diseases among MSM in China, we assumed that 40.1%, 25.1%, 4.8%, 30.0%, and 1.5/100,000 of them were at state S, L, G, H, and A, respectively, from the beginning of our model simulation (43–45). The initial proportions of MSM at states L and H did not differ among ages. We considered 37 HPV subtypes (low-risk subtypes: HPV-6, 11, 26, 34, 40, 42, 43, 44, 53, 54, 55, 57, 61, 66, 67, 69, 70, 71, 72, 73, 81, 82, 83, 84; high-risk subtypes: HPV-16, 18, 31, 33, 35, 39, 45, 51, 52, 56, 58, 59, 68) to promise better accuracy of final infection outcomes. Apart from the deaths from anal cancer, natural deaths among MSM were taken into account. Besides, status A in the model was assumed to exclude the precancerous lesions of anal cancer.

#### 2.2.2 Age stratification

In view of the differences in sex frequency and sexual activeness level among individuals of various ages, MSM were divided into three age groups with cut-off points of 27 and 45 years (age groups, MSM aged <27, 27-45, and >45 years). Further, the incidence rates of any-type HPV infection were assumed to be different among these three groups. We assumed that the ages of MSM in each group follow uniform distribution, and simulated the growth of ages for a more solid result. MSM before the age of puberty (<9 years old) were excluded from all analyses. The criterion for the cut-off point selection refers to current recommendations of age-appropriate HPV vaccination for women, which indicate that adults aged 27-45 years might be at risk of new HPV infection and benefit from vaccination (1).

#### 2.2.3 Interventions

HPV vaccination was the primary pre-exposure preventative intervention in our model. We assumed that a bivalent vaccine could provide a 94.0% protection effect against the infection of its covered HPV subtypes (HPV-16, 18) (36), while the protection effect on other subtypes is 0%. Similarly, 90.4% protection effect against the infection of HPV-6, 11, 16, and 18, and 96.7% against the infection of HPV-6, 11, 16, 18, 31, 33, 45, 52, and 58 were provided by quadrivalent vaccine and nine-valent vaccine, respectively (37, 39), while no protective effect was supposed against their corresponding uncovered subtypes. MSM who were susceptible to HPV infection (S) would be vaccinated at a certain initial coverage and an annual vaccinating rate. The duration of protection from HPV vaccination was assumed to be lifelong.

Vaccination plans in this study were age-stratified to provide comprehensive recommendations. We considered combined vaccination strategies with various HPV vaccine categories instead of a single type of vaccine. Alternative vaccination strategies were built by allocating bivalent, quadrivalent, nine-valent, or no vaccine to each of the three age groups. A total of 64 vaccination strategies were involved in subsequent effectiveness and cost-effectiveness analyses, classified as a) one-group-targeted strategy: allocating one kind of vaccines to one of the three age groups; b) two-groups-targeted strategy: allocating bivalent, quadrivalent, or nine-valent to two of the age groups; c) full-arrangement strategy: allocating bivalent, quadrivalent, or nine-valent to all age groups. No vaccination was assumed as the baseline scenario.

### 2.3 Model simulation

The outcomes of the model included the existing number of HPV low-risk and high-risk infections, anogenital warts, anal cancers, and deaths from anal cancer. The prevented infections and deaths by vaccination compared with baseline were calculated to assess the effectiveness of vaccines. Total costs (incurred by anogenital warts treatment, anal cancer treatment, and vaccination), quality-adjusted life years (QALYs), and incremental cost-effectiveness ratios (ICERs, the gained QALYs divided by increased total costs) were obtained for the evaluation of cost-effectiveness. The discount rate (year-end) was set at an average of 0.03 to convert future values of costs into equivalent present values. A vaccination strategy is judged to be cost-effective when its estimated ICER is less than three times the gross domestic product (GDP) per capita, and it is considered a plan with high cost-effectiveness if the ICER is less than per capita GDP. Details about the outcome calculation are shown in Appendix.

The model was run 1000 times in each setting to narrow the errors from parameter selection. The medians and inter-quartile range (IQR) of all outcomes were estimated based on Monte Carlo simulation. The simulation period was set as 10 years. We implemented sensitivity analysis for the vaccination coverage (20%, 50%, 90%), annual vaccinating rate (10%, 20%, 30%, 40%), vaccine price (+25%, -25%, -50%, -75%) and discount rate (-25%, +25%), to assess the robustness of final results. All computations were performed by R 4.1.2 (R Foundation for Statistical Computing, Vienna, Austria).

## 3 Results

### 3.1 Baseline analysis

**Figure 2:**
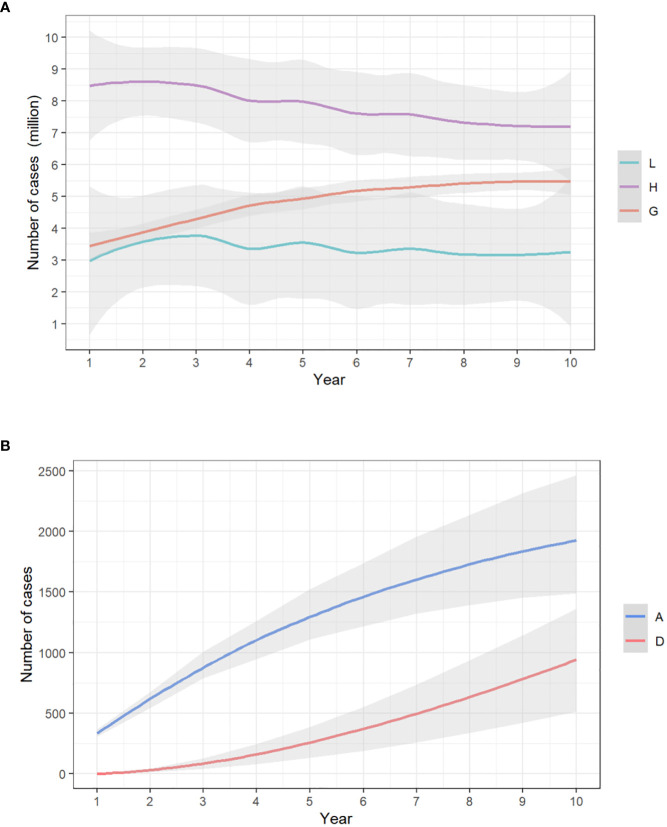
The yearly trends of **(A)** low-risk HPV, high-risk HPV, and anogenital warts infections/**(B)** anal cancer infections and deaths from anal cancer, with no vaccination. The x-axis relates to the simulation period (10 years in total). The y-axis relates to the number of cases in each model compartment. L, low-risk HPV infection; H, high-risk HPV infection; G, anogenital warts; A, anal cancer; D, death from anal cancer.

The model showed that with no vaccination, in 10 years, the existing cases of anogenital warts would reach 5,464,225 (IQR, 4,685,708-6,174,175); that of anal cancer would reach 1,922.95 (1,716.56-2,119.93), resulting in 940.55 (732.27-1,141.87) deaths. In general, the 10-year simulation yielded a total of 11,235,709 (10,198,499-12,385,242) cumulative anogenital warts and 2,863.50 (2,722.13-2,998.67) anal cancers. The annual new infections peaked at 779,891 (606,671.60-944,629.90) at year 2 for anogenital warts, and 157.54 (128.30-186.39) at year 2 for anal cancer. The trends of yearly infections are shown in **Figure 2**.

### 3.2 Effectiveness of vaccination strategies

#### 3.2.1 One-group-targeted strategy

**Figure 3:**
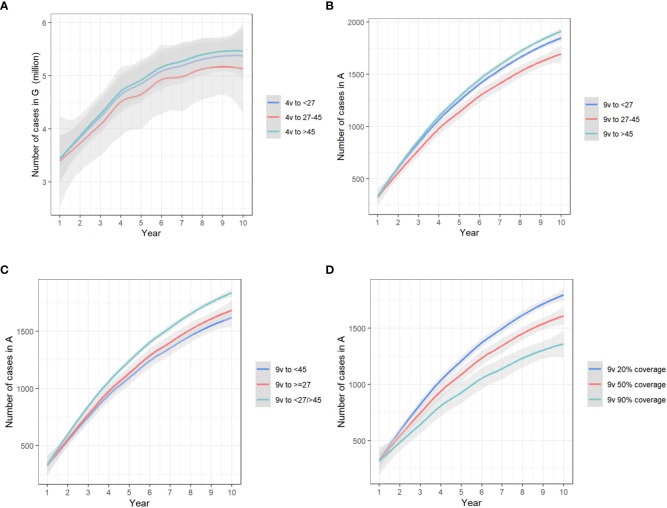
The trends of yearly infections in **(A)** state G, with quadrivalent vaccines allocated to different groups in the one-group-targeted scenario/**(B)** state A, with nine-valent vaccines allocated to different groups in the one-group-targeted scenario/**(C)** state A, with nine-valent vaccines in the two-group-targeted scenario/**(D)** state A, with nine-valent vaccines targeted at all groups under different coverages. The x-axis relates to the simulation period (10 years total). The y-axis relates to the number of cases in different model compartments. State G, anogenital warts; state A, anal cancer. 4v, quadrivalent vaccines; 9v, nine-valent vaccines.

Under 50% vaccination coverage among susceptible MSM in one age group, compared with baseline, bivalent HPV vaccines had no effect in the prevention of anogenital warts, even though a maximum of 73.91(66.77-80.24) anal cancer cases could be avoided; the prevented cases of anogenital warts were maximized (median, 378,458.92, IQR, 314,785.72-437,167.42) with quadrivalent HPV vaccines allocated to MSM aged 27-45 years; that of anal cancer (231.44, 208.29-252.58) and deaths (104.80, 81.18-127.75) was maximized when providing nine-valent vaccines to MSM aged 27-45 years. (**Figures 3A, B**)

#### 3.2.2 Two-group-targeted strategy

Under 50% vaccination coverage among susceptible MSM in two age groups, compared with baseline, the prevented cases of anogenital warts were maximized (480,787.99, 405,437.58-553,663.48) with quadrivalent HPV vaccines allocated to MSM aged <27 and 27-45 years; that of anal cancer (305.69, 275.33-333.30) and deaths (136.60, 105.76-166.55) was maximized when providing nine-valent vaccines to MSM aged < 45 years. (**Figure 3C**)

#### 3.2.3 Full-arrangement strategy

Under 50% coverage among susceptible MSM at all ages, compared with baseline, bivalent HPV vaccination could prevent 5.15% (5.03%-5.28%) anal cancers and 4.52% (4.49%-4.54%) deaths, but marginally increased the quantity of anogenital warts (8.11‰, 5.62‰-11.89‰); quadrivalent vaccination prevented 9.08% (8.46%-9.50%) anogenital warts, 2.44% (2.29%-2.59%) anal cancers and 2.46% (2.41%-2.52%) deaths; and nine-valent vaccination could reduce 6.99% (6.36%-7.45%) anogenital warts, 16.44% (16.27%-16.62%) anal cancers and 15.01% (14.93%-15.05%) deaths. Under the same assumption on vaccination coverage, the prevented cumulative cases of anogenital warts were maximized (496,080.68, 418,945.15-570,872.88) with quadrivalent HPV vaccines allocated to all MSM age groups; that of anal cancer (316.11, 284.65-344.70) and deaths (141.14, 109.26-172.08) were maximized when providing nine-valent vaccines to all groups. (**Table 2**)

**Table 2:** The prevented cases and deaths of HPV-related diseases of age-stratified HPV vaccination strategies among MSM in China, compared with no vaccination.

Vaccination strategies^a^			Prevented anogenital warts (median [IQR])	Prevented anal cancers (median [IQR])	Prevented deaths from anal cancer (median [IQR])
<27	27-45	>45			
Strategies preventing the most anogenital warts					
One-group-targeted strategies					
0	4	0	378,458.92 [314,785.72, 437,167.42]	33.63 [29.49, 37.57]	15.61 [12.33, 18.92]
Two-group-targeted strategies					
4	4	0	480,787.99 [405,437.58, 553,663.48]	44.97 [39.71, 49.92]	22.12 [17.44, 26.87]
9	4	0	459,992.91 [386,887.89, 530,320.03]	107.88 [97.02, 117.56]	47.41 [36.88, 57.70]
Full-arrangement strategies					
4	4	4	496,080.68 [418,945.15, 570,872.88]	46.99 [41.54, 52.14]	23.14 [18.23, 28.11]
4	4	9	494,267.64 [417,365.36, 569,060.08]	55.39 [49.26, 61.09]	26.66 [21.03, 32.47]
9	4	4	475,285.60 [400,409.88, 548,298.84]	109.90 [98.82, 119.73]	48.43 [37.67, 58.94]
Strategies preventing the most anal cancers and deaths					
One-group-targeted strategies					
0	9	0	286,959.37 [232,059.73, 332,853.55]	231.44 [208.29, 252.58]	104.80 [81.18, 127.75]
Two-group-targeted strategies					
9	9	0	368,493.36 [304,160.39, 422,998.40]	305.69 [275.33, 333.30]	136.60 [105.76, 166.55]
2	9	0	259,256.07 [207,993.55, 301,422.87]	253.39 [228.01, 276.43]	114.60 [88.74, 139.70]
Full-arrangement strategies					
9	9	9	381,973.01 [316,108.32, 439,370.04]	316.11 [284.65, 344.70]	141.14 [109.26, 172.08]
9	9	2	366,064.35 [301,831.89, 420,016.23]	308.95 [278.24, 336.85]	138.03 [106.86, 168.28]
9	9	4	383,786.05 [317,676.35, 441,279.65]	307.71 [277.11, 335.56]	137.62 [106.55, 167.80]

HPV, human papillomavirus; MSM, men who have sex with men; IQR: inter-quartile range.

Statistically significant increase in the number of anogenital warts is indicated if the upper band of IQR of prevented cases is less than 0; no positive or negative effect of vaccination strategies is suggested if the IQR covers 0.

Vaccination strategies: "<27", "27-45", and ">45" represent the three MSM groups stratified by age (MSM aged <27, 27-45, and >45 years, respectively). Number "0" represents no vaccine. Numbers "2", "4" and "9" represent the bivalent vaccine, quadrivalent vaccine, and nine-valent vaccine, respectively. Each row is an alternative allocation strategy of the three vaccine categories. For example, the line filled with "2", "4", and "2" means allocating bivalent vaccine to MSM aged <27 and >45 years, and quadrivalent vaccine to those aged 27-45 years.

With nine-valent vaccines, when the vaccination coverage rose from 50% to 90% for all age groups, 6.01% (5.44%-6.44%) anogenital warts, 15.74% (15.55%-15.95%) anal cancers and 14.12% (14.04%-14.17%) deaths from anal cancer could be further prevented. When the vaccination coverage was raised from 50% to 90% separately in MSM aged <27 years, 1.28% (1.26%-1.31%) anogenital warts, 3.70% (3.66%-3.75%) anal cancers and 3.18% (3.15%-3.20%) deaths could be prevented. With the same separate increase of vaccination coverage among MSM aged 27-45 years, 4.52% (3.94%-4.92%) anogenital warts, 11.52% (11.36%-11.69%) anal cancers and 10.49% (10.44%-10.52%) deaths could be avoided, and that separate increase among MSM aged >45 years would only reduce 2.12‰ (2.08‰-2.17‰) anogenital warts, 5.19‰ (5.13‰-5.26‰) anal cancers and 4.54‰ (4.50‰-4.56‰) deaths. Conversely, if reducing the vaccination coverage from 50% to 20% for all age groups, the cases of anogenital warts, cases of anal cancer, and deaths would be increased by 4.51% (4.08%-4.83%), 11.80% (11.66%-11.96%) and 10.59% (10.53%-10.63%), respectively. (**Figure 3D**)

### 3.3 Cost-effectiveness of vaccination strategies

#### 3.3.1 One-group-targeted strategy

Compared with baseline, under 50% vaccination coverage, allocating bivalent vaccines to MSM aged >45 years was the most cost-saving strategy (total cost increased: 36.98 million USD, 36.33-37.63). Vaccinating MSM aged 27-45 years with quadrivalent vaccines improved the most QALY (quality-adjusted life year, 34,087.30, 28,359.07-39,367.77). The lowest ICER value (29,871.06 USD/QALY, 26,993.53-32,761.46) was reached when allocating quadrivalent vaccines to MSM aged 27-45 years.

#### 3.3.2 Two-group-targeted strategy

Compared with baseline, under 50% vaccination coverage, allocating bivalent vaccines to MSM aged <27 and >45 years was the most cost-saving strategy (total cost increased: 291.09 million USD, 283.87-298.31). Vaccinating MSM <27 years old and those aged 27-45 years improved the most QALY (43,306.92, 36,523.99-49,860.28). The lowest ICER value (30,575.34 USD/QALY, 27,686.32-33,477.29) was reached when allocating quadrivalent vaccines to MSM aged 27-45 and >45 years.

#### 3.3.3 Full-arrangement strategy

**Figure 4:**
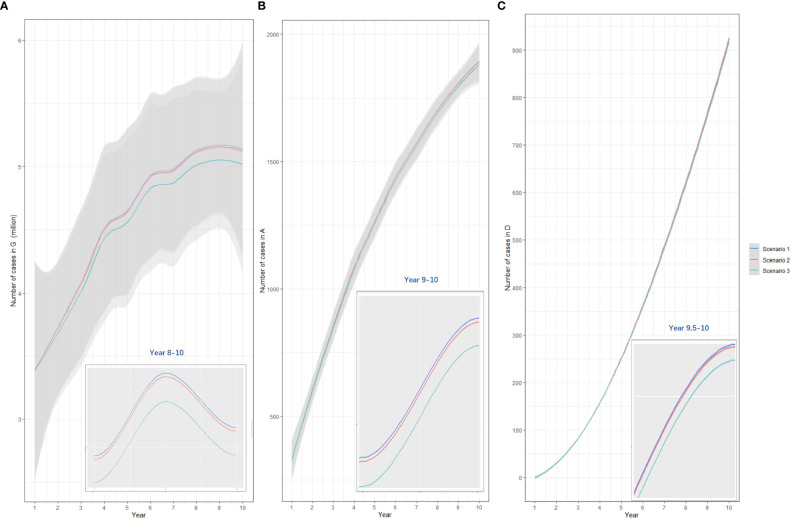
The comparison of yearly anogenital warts infections **(A)**, anal cancers **(B)**, and deaths from anal cancer **(C)** under three vaccination scenarios. Scenario 1: allocating quadrivalent vaccines to MSM aged 27-45 years; scenario 2: allocating quadrivalent vaccines to MSM aged ≥27 years, scenario 3: allocating quadrivalent vaccines to MSM at all ages. The x-axis relates to the simulation period (10 years total). The y-axis relates to the number of cases in different model compartments. G, anogenital warts; A, anal cancer; D, death from anal cancer.

**Figure 5:**
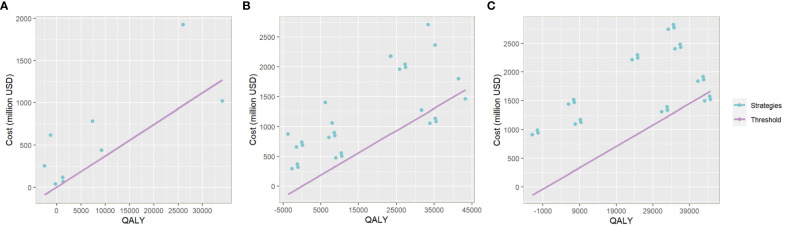
The plot of QALY and Cost of each vaccination strategy in the one-group-targeted **(A)**, two-group-targeted **(B)**, and full-arrangement **(C)** scenarios. The x-axis relates to the gained QALY of each vaccination strategy compared with the baseline scenario. The y-axis relates to the total cost increase of each strategy compared with the baseline. The slope of the line in purple is three times China’s per capita GDP. Plots below the line represent strategies with cost-effectiveness.

Compared with baseline, under 50% vaccination coverage, allocating bivalent vaccines to all MSM age groups was the most cost-saving strategy (total cost increased: 910.30 million USD, 891.93-928.68). Vaccinating MSM <27 years old, MSM aged 27-45 years and those >45 years with quadrivalent vaccines improved the most QALY (quality-adjusted life year) (44,684.90, 37,740.83-51,416.49). The lowest ICER value (34,098.09 USD/QALY, 31,146.54-37,062.88) was reached when allocating quadrivalent vaccines to MSM in all age groups, and this value has fallen below three times the per capita GDP (per capita gross domestic product, 12420 USD in the year 2022) in China. Under such a vaccination strategy, 18,897,954 doses of quadrivalent vaccines, and a total of 7,783.00 (6,153.96-9,404.89) million USD would be consumed, resulting in a total of 28,400,716 (28,343,914-28,465,416) QALY among MSM. (**Table 3**) The comparison of yearly infection trends with the most cost-effective strategies determined in all scenarios is shown in **Figure 4**. The cost-effectivenesses of all vaccination strategies in the three separate scenarios are shown in **Figure 5**.

**Table 3:** The gained QALY, increased cost and ICER of age-stratified HPV vaccination strategies among MSM in China, compared with no vaccination.

Vaccination strategies^a^			QALY gained (median [IQR])	Total cost increased (million USD, median [IQR])	ICER (1000 USD/QALY, median [IQR])
<27	27-45	>45			
Strategies increasing the most QALY					
One-group-targeted strategies					
0	4	0	34,087.30 [28,359.07, 39,367.77]	1,018.22 [920.14, 1,116.75]	29.87 [26.99, 32.76]
Two-group-targeted strategies					
4	4	0	43,306.92 [36,523.99, 49,860.28]	1,457.53 [1,330.01, 1,585.62]	33.66 [30.71, 36.61]
9	4	0	41,480.33 [34,913.76, 47,812.63]	1,800.49 [1,677.98, 1,923.55]	43.41 [40.45, 46.37]
Three-group-targeted strategies					
4	4	4	44,684.90 [37,740.83, 51,416.49]	1,523.67 [1,391.78, 1,656.15]	34.10 [31.15, 37.06]
4	4	9	44,527.88 [37,605.44, 51,261.67]	1,574.17 [1,442.75, 1,706.19]	35.35 [32.40, 38.32]
4	4	2	43,090.76 [36,340.46, 49,625.08]	1,494.51 [1,367.65, 1,621.94]	34.68 [31.74, 37.64]
Strategies increasing the least cost					
One-group-targeted strategies					
0	0	2	-216.16 [-241.83, -189.01]	36.98 [36.33, 37.63]	/
Two-group-targeted strategies					
2	0	2	-2,692.83 [-3,055.41, -2,348.21]	291.09 [283.87, 298.31]	/
2	0	4	-1,098.68 [-1,261.05, -931.09]	320.24 [318.04, 322.43]	/
Three-group-targeted strategies					
2	0	4	-1,098.68 [-1,261.05, -931.09]	320.24 [318.04, 322.43]	/
2	2	4	-2,319.76 [-3,987.22, -1,036.81]	939.46 [926.08, 952.77]	/
2	2	9	-2,476.78 [-4,146.38, -1,211.59]	989.96 [976.12, 1003.73]	/
Strategies with cost-effectiveness (sort all the scenarios by ICER)^b^					
0	4	0	34,087.30 [28,359.07, 39,367.77]	1,018.22 [920.14, 1,116.75]	29.87 [26.99, 32.76]
0	4	4	35,465.28 [29,577.88, 40,863.80]	1,084.36 [981.90, 1,187.28]	30.58 [27.69, 33.48]
0	4	2	33,871.13 [28,160.24, 39,115.12]	1,055.20 [957.77, 1,153.07]	31.15 [28.28, 34.04]
0	4	9	35,308.25 [29,452.22, 40,683.88]	1,134.87 [1,032.87, 1,237.32]	32.14 [29.25, 35.04]
4	4	0	43,306.92 [36,523.99, 49,860.28]	1,457.53 [1,330.01, 1,585.62]	33.66 [30.71, 36.61]
4	4	4	44,684.90 [37,740.83, 51,416.49]	1,523.67 [1,391.78, 1,656.15]	34.10 [31.15, 37.06]
4	4	2	43,090.76 [36,340.46, 49,625.08]	1,494.51 [1,367.65, 1,621.94]	34.68 [31.74, 37.64]
4	4	9	44,527.88 [37,605.44, 51,261.67]	1,574.17 [1,442.75, 1,706.19]	35.35 [32.40, 38.32]

HPV, human papillomavirus; MSM, men who have sex with men; IQR: inter-quartile range; QALY, quality-adjusted life years; ICER, incremental cost-effectiveness ratio.

The ICER values of cost-effective strategies are less than three times the per capita GDP (gross domestic product) in China. "/" indicates that the calculation of ICER is meaningless, as no gained QALY is detected.

a Vaccination strategies: "<27", "27-45", and ">45" represent the three MSM groups stratified by age (MSM aged <27, 27-45, and >45 years, respectively). Number "0" represents no vaccine. Numbers "2", "4" and "9" represent bivalent vaccine, quadrivalent vaccine, and nine-valent vaccine, respectively. Each row is an alternative allocation strategy of the three vaccine categories. For example, the line filled with "2", "4", "2" means allocating bivalent vaccine to MSM aged <27 and >45 years, and quadrivalent vaccine to those aged 27-45 years.

b All strategies with cost-effectiveness are listed.

### 3.4 Sensitivity analysis

When the overall vaccination coverage declined from 50% to 20% or rose from 50% to 90% consistently among all the vaccinated age groups, the ICERs of alternative vaccine allocations remained stable. Based on the most cost-effective vaccination strategy in the one-group-targeted scenario, when the annual vaccinating rate among MSM aged 27-45 years increased from 0% to 10%, the ICER would rise to 42,975.92 USD/QALY (40,373.25-45,590.25). That value would fall below the threshold (three times China’s per capita GDP) when elevating the vaccination rate to 20% (37,234.35 USD/QALY, 34,736.25-39,743.65). Based on the most cost-effective vaccination strategy in the two-group-targeted scenario, when the annual vaccination rate among MSM aged ≥27 years increased from 0% to 10%, the ICER would rise to 41,018.25 USD/QALY (38,437.79-43,610.30), and that value would also fall below the threshold if increasing the vaccinating rate to 20% (35,116.42 USD/QALY, 32,637.44-37,606.52). A similar trend was detected for the most cost-effective vaccination strategy in the full-arrangement scenario, except that the ICER value (40,060.47 USD/QALY, 37,523.32-42,609.02) still exceeded the threshold under 50% vaccination coverage and 20% vaccinating rate. A higher annual vaccination rate was expected to further contain the ICER (vaccination rate, 30%, ICER, 33,521.75 USD/QALY, 31,040.73-36,013.92). Simultaneously, in the case with a 0% vaccinating rate, quadrivalent vaccines to all groups, and 50% coverage, when the vaccine price decreased by 25%, the ICER was reduced to 22,950.74 USD/QALY (19,999.19-25,915.53). When the vaccine price decreased by 60%, the ICER was reduced to 7,344.44 USD/QALY (4,392.89-10,309.23), indicating good cost-effectiveness taking China’s per capita GDP as a threshold. When the discount rate rose by 25%, the value of ICER was reduced to 33,068.32 USD/QALY (30,205.91-35,943.58), and the ICER would increase to 35,183.18 USD/QALY (32,137.70-38,242.32) if the discount rate decreased by 25%. (**Table 4**)

**Table 4:** The ICER values of different HPV vaccination strategies in sensitivity analyses.

	Strategy		
	4v to 27-45	4v to 27-45/>45	4v to <27/27-45/>45
Vaccination coverage^a^			
20%/90% (all those vaccinated)	Stable	Stable	Stable
20% (27-45) 50% (others)	Stable	31,534.78 [28,630.11, 34,452.47]	37,665.73[34,651.70, 40,693.30]
90% (27-45) 50% (others)	Stable	30,269.20 [27,385.18, 33,166.13]	32,496.11 [29,572.61, 35,432.70]
Annual vaccinating rate			
10%	42,975.92 [40,373.25, 45,590.25]	41,018.25 [38,437.79, 43,610.30]	45,873.26 [43,239.08, 48,519.27]
20%	37,234.35 [34,736.25, 39,743.65]	35,116.42 [32,637.44, 37,606.52]	40,060.47 [37,523.32, 42,609.02]
30%	30,319.69 [27,891.83, 32,758.44]	28,652.93 [26,236.89, 31,079.83]	33,521.75 [31,040.73, 36,013.92]
40%	24,399.23 [22,034.98, 26,774.11]	23,253.04 [20,892.52, 25,624.16]	28,022.84 [25,590.24, 30,466.37]
Vaccine price^b^			
+25%	39,895.59 [37,018.07, 42,785.99]	40,786.23 [37,897.21, 43,688.18]	45,245.44 [42,293.89, 48,210.24]
-25%	19,846.53 [16,969.00, 22,736.93]	20,364.44 [17,475.43, 23,266.40]	22,950.74 [19,999.19, 25,915.53]
-50%	9,822.00 [6,944.47, 12,712.40]	10,153.55 [7,264.54, 13,055.51]	11,803.38 [8,851.83, 14,768.175]
-60%	5,812.18 [2,934.66, 8,702.58]	6,069.20 [3,180.18, 8,971.15]	7,344.44 [4,392.89, 10,309.23]
Discount rate^b^			
-25%	30,821.64 [27,852.54, 33,804.02]	31,548.32 [28,567.37, 34,542.63]	35,183.18 [32,137.70, 38,242.32]
+25%	28,968.95 [26,178.32, 31,772.06]	29,651.95 [26,850.19, 32,466.27]	33,068.32 [30,205.91, 35,943.58]

"<27", "27-45", and ">45" represent the three MSM groups stratified by age (MSM aged <27, 27-45, and >45 years, respectively); "4v" under "Strategy" represents quadrivalent HPV vaccine. The background vaccination coverage is 50%.

The ICER values are shown as median [IQR].

ICER, incremental cost-effectiveness ratio; HPV, human papillomavirus; IQR, inter-quartile range.

a The ICER values remained stable when consistently improving the overall coverage in all vaccinated groups.

b "-" and "+" denote decrease and increase on the original basis (shown in **Table 1**), respectively.

## 4 Discussion

Our model showed that among MSM in China, HPV vaccination could ease the burden of anogenital warts and anal cancer to some extent. Quadrivalent vaccines showed the most effectiveness in the prevention of anogenital warts overall, and nine-valent vaccines avoided the most anal cancer cases. Primarily vaccinating MSM aged 27-45 years was generally the optimal choice. With 50% coverage among all susceptible MSM, the lowest ICER (34,098.09 USD/QALY, 31,146.54-37,062.88) was reached when allocating quadrivalent vaccines to all age groups. An additional annual vaccination rate of at least 20% and a decrease IN vaccine price could further reduce the ICER.

In consistence with our findings, Winer et al. also detected that quadrivalent vaccines were effective in preventing genital HPV infections in young MSM, with an efficacy of around 85% (46). Nine-valent HPV vaccination was suggested as an effective pre-exposure prophylaxis intervention against anal HPV infection, as approximately 73% of MSM in their survey had ≥1 nine-valent vaccine HPV subtypes (47). The bivalent vaccine was less effective in controlling HPV-related diseases among MSM, as it made no difference in the reduction of anogenital warts cases. However, it was the most cost-saving. In reality, MSM may prefer bivalent vaccines as their acceptability of HPV vaccines was highly associated with price, which was shown in a cross-sectional study targeted at MSM in Hong Kong (48). Li et al. found that only 2.5% of MSM in mainland China were willing to afford more than 295 USD for HPV vaccination (49). If primarily considering the affordability of vaccines, and promoting bivalent vaccines among MSM, the medical treatment for patients with anogenital warts should be improved to ensure their quality of life.

The lower price in comparison with nine-valent vaccines, and the higher protective effect against anogenital warts compared with bivalent vaccines have made quadrivalent vaccines a choice with higher cost-effectiveness. As shown in a systematic review and meta-analysis, the involved studies for MSM at various ages consistently demonstrated the cost-effectiveness of quadrivalent HPV vaccines compared with no vaccination (50). However, most of the vaccination strategies (87.30%) were not cost-effective in China, and good cost-effectiveness was not yet achieved. Different conclusions were drawn in the USA, where the estimated ICER of quadrivalent vaccines was only $15,290 per QALY when MSM were vaccinated at 12 years old under 10% coverage, and less than the per capita GDP under most scenarios (approximately 95%) (22). Actions should be taken in China to increase the willingness-to-pay threshold among MSM. Besides, HPV vaccines tended to be more cost-effective if only considering diseases related to high-risk HPV infection. A cross-sectional study indicated that among MSM in Shenyang, China, bivalent vaccines were cost-effective for the prevention of anal cancer (51). Vaccines providing protection against more low-risk HPV subtypes should be developed to fit in with potential vaccination promotion among MSM.

Appropriate vaccination age is also of critical importance to ensure maximum cost-effectiveness. Corresponding with our results, Deshmukh et al. also recommended the primary expansion of current vaccination guidelines to MSM aged ≥27, and the younger were endowed with higher priority (32). The main cause may be the high average level of sexual activity and infection risk among MSM in this age group. Glick et al. found that up to 72% of 35- to 39-year-olds MSM in 4 population-based surveys formed a new partnership during the previous year, while among those aged less than 18 years generally fewer sex partners were acknowledged (52). Ethe et al. showed that the adjusted odds-ratio of anal HPV prevalence comparing MSM aged <30 years and >45 years was 2.714 (53). This evidence have indicated the importance of risk classification during vaccination promotion. The potential individual infection risk of HPV should be adequately evaluated so that more targeted vaccination is carried out, especially in the case of insufficient vaccine supply.

Interventions facilitating the decline of ICER should be considered. For instance, an additional annual vaccination rate was essential to increase the cost-effectiveness. A good cost-effectiveness of vaccination strategies could also be achieved by a 60% decrease in vaccine price. Similar results were obtained in studies in various regions. A study in England also found the decrease in price beneficial, as bivalent vaccination was not cost-effective except when reaching a price which was £41/dose cheaper than the quadrivalent vaccine (54). Researchers have suggested that low- and middle-income countries should firstly adjust HPV vaccine prices to improve the cost-effectiveness (55). Manufacturers in China should accelerate the development of domestic vaccines to reduce the cost of logistics, and partially subsidized vaccination should be further considered to release the burden of production costs. Reducing the dose each vaccination could also make sense. The World Health Organization (WHO) has recommended one-dose HPV vaccination in 2022 (56). As shown in a projected randomized trial conducted for women in Kenya, a reduction in HPV dose vaccination could effectively improve the vaccine coverage, apart from the savings of cost (57). Long-term clinical data on the efficacy of reduced dose vaccination among MSM are demanded to implement the plans. Furthermore, the reduction in vaccine price and dose vaccination may accelerate the expansion of HPV vaccines to men, while targeted vaccination towards MSM should be emphasized as HPV vaccines was rarely found to be cost-effective in heterosexual men (58).

Our study is one of the first studies exploring the effectiveness and cost-effectiveness of combined HPV vaccination strategies among age-stratified MSM in China. The primary outcomes could provide practical references for the improvement of HPV vaccination plans, as well as the promotion of HPV vaccines targeted at high-risk groups. However, there exist several limitations. First, due to the lack of detailed data on anal HPV’s development among MSM in China, precancerous lesions of anal cancer (low/high-grade anal squamous intraepithelial neoplasia) were not involved as compartments in the model, and chronologically spaced parameters may cause the risk of imbalance. Second, longitudinal data on anogenital warts and anal cancer prevalences were also severely absent among MSM in China, which made it difficult to achieve model calibration. More attention on HPV-related diseases among MSM should be attracted, and cohort studies with yearly follow-up data are expected in China. Third, HPV infections beyond 37 general subtypes and the cross-infection between anogenital warts and anal cancer was not considered, which may lead to an underestimation of vaccination effectiveness. Finally, vaccination adherence was not included as a parameter in our simulation. An individual-based model should be developed to better evaluate the impact of personal adherence level.

In conclusion, HPV vaccines can effectively reduce the prevalence of related diseases and deaths caused by anal cancer among MSM in China, especially quadrivalent vaccines for anogenital warts and nine-valent vaccines for anal cancer. Quadrivalent vaccines were always the most cost-effective, and MSM aged 27-45 years was the optimal age group for vaccination. However, none of the HPV vaccination plans indicated good cost-effectiveness taking China’s per capita GDP as a threshold. The improvement of initial vaccination coverage could prevent more infections of HPV-related diseases while making no difference on the ICER. Annual vaccination at an ideal level and appropriate adjustment of vaccine price is necessary to further improve the cost-effectiveness.

## Data availability statement

The original contributions presented in the study are included in the article/**Supplementary Material**. Further inquiries can be directed to the corresponding author.

## Author contributions

HZ and Y-FL conceived the idea and protocol. YWL and Y-FL built the model, collected the data, finalized the analysis, interpreted the findings, and wrote the manuscript. All authors critically reviewed and approved the final manuscript.

## References

[1] Kamolratanakul S Pitisuttithum P. Human papillomavirus vaccine efficacy and effectiveness against cancer. Vaccines (Basel) (2021) 9:1413. doi: 10.3390/vaccines9121413 34960159 PMC8706722

[2] Tommasino M. The human papillomavirus family and its role in carcinogenesis. Semin Cancer Biol (2014) 26:13–21. doi: 10.1016/j.semcancer.2013.11.002 24316445

[3] Egawa N Doorbar J. The low-risk papillomaviruses. Virus Res (2017) 231:119–27. doi: 10.1016/j.virusres.2016.12.017 28040475

[4] Crosbie EJ Einstein MH Franceschi S Kitchener HC. Human papillomavirus and cervical cancer. Lancet (2013) 382:889–99. doi: 10.1016/s0140-6736(13)60022-7 23618600

[5] Sammarco ML Ucciferri C Tamburro M Falasca K Ripabelli G Vecchiet J. High prevalence of human papillomavirus type 58 in HIV infected men who have sex with men: a preliminary report in central Italy. J Med Virol (2016) 88:911–4. doi: 10.1002/jmv.24406 26467111

[6] Senkomago V Henley SJ Thomas CC Mix JM Markowitz LE Saraiya M. Human papillomavirus-attributable cancers - united states, 2012-2016. MMWR Morb Mortal Wkly Rep (2019) 68:724–8. doi: 10.15585/mmwr.mm6833a3 PMC6705893 31437140

[7] Grulich AE Jin F Conway EL Stein AN Hocking J. Cancers attributable to human papillomavirus infection. Sex Health (2010) 7:244–52. doi: 10.1071/sh10020 20719211

[8] Lu Y Li P Luo G Liu D Zou H. Cancer attributable to human papillomavirus infection in China: burden and trends. Cancer (2020) 126:3719–32. doi: 10.1002/cncr.32986 32484937

[9] Liu H Yang H Li X Wang N Liu H Wang B. Men who have sex with men and human immunodeficiency virus/sexually transmitted disease control in China. Sex Transm Dis (2006) 33:68–76. doi: 10.1097/01.olq.0000187266.29927.11 16432476

[10] Ucciferri C Tamburro M Falasca K Sammarco ML Ripabelli G Vecchiet J. Prevalence of anal, oral, penile and urethral human papillomavirus in HIV infected and HIV uninfected men who have sex with men. J Med Virol (2018) 90:358–66. doi: 10.1002/jmv.24943 28906006

[11] Zou H Tabrizi SN Grulich AE Hocking JS Bradshaw CS Cornall AM. Site-specific human papillomavirus infection in adolescent men who have sex with men (HYPER): an observational cohort study. Lancet Infect Dis (2015) 15:65–73. doi: 10.1016/s1473-3099(14)70994-6 25435055

[12] Donà MG Vescio MF Latini A Giglio A Moretto D Frasca M. Anal human papillomavirus in HIV-uninfected men who have sex with men: incidence and clearance rates, duration of infection, and risk factors. Clin Microbiol Infect (2016) 22:1004.e1001–1004.e1007. doi: 10.1016/j.cmi.2016.08.011 27585942

[13] Tian T Mijiti P Bingxue H Fadong Z Ainiwaer A Guoyao. Prevalence and risk factors of anal human papillomavirus infection among HIV-negative men who have sex with men in urumqi city of xinjiang uyghur autonomous region, China. PloS One (2017) 12:e0187928. doi: 10.1371/journal.pone.0187928 29141014 PMC5687769

[14] Mladěnka A Sláma J. Vaccination against HPV and view of new possibilities. Ceska Gynekol (2018) 83:218–25. 30764623

[15] Gong L Ji HH Tang XW Pan LY Chen X Jia YT. Human papillomavirus vaccine-associated premature ovarian insufficiency and related adverse events: data mining of vaccine adverse event reporting system. Sci Rep (2020) 10:10762. doi: 10.1038/s41598-020-67668-1 32612121 PMC7329819

[16] Hao J Jiandong W. How to effectively control cervical cancer? Dev Cancers (2021) 19:1964.

[17] Chesson HW Meites E Ekwueme DU Saraiya M Markowitz LE. Cost-effectiveness of nonavalent HPV vaccination among males aged 22 through 26 years in the united states. Vaccine (2018) 36:4362–8. doi: 10.1016/j.vaccine.2018.04.071 PMC6745692 29887325

[18] Nan W Qi L Junhong L Yamin W Chao M Canjun Z. The current status of inclusion of vaccines from the 194 member states of the world health organization into the national immunization program. China Vaccines Immunization (2021) 27:214–20. doi: 10.19914/j.cjvi.2021037

[19] Bonanni P Faivre P Lopalco PL Joura EA Bergroth T Varga S. The status of human papillomavirus vaccination recommendation, funding, and coverage in WHO Europe countries (2018-2019). Expert Rev Vaccines (2020) 19:1073–83. doi: 10.1080/14760584.2020.1858057 33267673

[20] Colzani E Johansen K Johnson H Pastore Celentano L. Human papillomavirus vaccination in the European Union/European economic area and globally: a moral dilemma. Euro Surveill (2021) 26:2001659. doi: 10.2807/1560-7917.Es.2021.26.50.2001659 34915976 PMC8728487

[21] Diagnosis IV. Men are expected to be vaccinated with domestic nine-valent HPV vaccine (2022). Available at: https://mp.weixin.qq.com/s?:biz=MzU1MTc5ODI2OQ==&mid=2247646665&idx=1&sn=6c498a817192ddb15b5adde8aa62b3c7&chksm=fb87295bccf0a04d5ddbd5b471d950368b4e76b1dd93c3c383073bfa9c7d9c50b3b31b02b559&scene=27.

[22] Kim JJ. Targeted human papillomavirus vaccination of men who have sex with men in the USA: a cost-effectiveness modelling analysis. Lancet Infect Dis (2010) 10:845–52. doi: 10.1016/s1473-3099(10)70219-x PMC3982926 21051295

[23] Zhang L Regan DG Ong JJ Gambhir M Chow EPF Zou H. Targeted human papillomavirus vaccination for young men who have sex with men in Australia yields significant population benefits and is cost-effective. Vaccine (2017) 35:4923–9. doi: 10.1016/j.vaccine.2017.07.078 28789853

[24] Hu M Xu C Wang J. Spatiotemporal analysis of men who have sex with men in mainland China: social app capture-recapture method. JMIR Mhealth Uhealth (2020) 8:e14800. doi: 10.2196/14800 32012086 PMC7007599

[25] Wei L Chen L Zhang H Yang Z Liu S Tan W. Relationship between gay app use and HIV testing among men who have sex with men in shenzhen, China: a serial cross-sectional study. BMJ Open (2019) 9:e028933. doi: 10.1136/bmjopen-2019-028933 PMC6721534 31446409

[26] China, N. B. o. S. o. Statistical year book (2022) (2022). Available at: http://www.stats.gov.cn/.

[27] Tuo XQ. Intermediary analysis of persistent HPV infection and its influencing factors in men who have sex with men. Urumqi, Xinjiang: Xinjiang Medical University (2019).

[28] Gong Z Zhang ZL Yeledan MH Tuo XQ Chen Z Guliya HLL. Analysis of potential categories of human papillomavirus 16/18 infection risk in men who have sex with men. China Gen Med (2019) 22:3549–53.

[29] Mao SS. Epidemic characteristics and influencing factors of HIV, syphilis and herpes simplex virus type 2 among men who have sex with men in shenzhen. Hengyang, Hubei: Nanhua University (2021).

[30] Zhou Y Zhou X Lin YF Luo G Lu Y Wang Z. Incidence, persistence, and clearance of anal human papillomavirus among men who have sex with men in China: an observational cohort study. Pathogens (2022) 11:314. doi: 10.3390/pathogens11030314 35335637 PMC8949987

[31] Arima Y Winer RL Feng Q Hughes JP Lee SK Stern ME. Development of genital warts after incident detection of human papillomavirus infection in young men. J Infect Dis (2010) 202:1181–4. doi: 10.1086/656368 20812849

[32] Deshmukh AA Chiao EY Das P Cantor SB. Clinical effectiveness and cost-effectiveness of quadrivalent human papillomavirus vaccination in HIV-negative men who have sex with men to prevent recurrent high-grade anal intraepithelial neoplasia. Vaccine (2014) 32:6941–7. doi: 10.1016/j.vaccine.2014.10.052 PMC4254641 25444820

[33] Zhang Z Ling X Liu L Xi M Zhang G Dai J. Natural history of anal papillomavirus infection in HIV-negative men who have sex with men based on a Markov model: a 5-year prospective cohort study. Front Public Health (2022) 10:891991. doi: 10.3389/fpubh.2022.891991 35646789 PMC9130828

[34] Machalek DA Poynten M Jin F Fairley CK Farnsworth A Garland SM. Anal human papillomavirus infection and associated neoplastic lesions in men who have sex with men: a systematic review and meta-analysis. Lancet Oncol (2012) 13:487–500. doi: 10.1016/s1470-2045(12)70080-3 22445259

[35] Zhu FC Chen W Hu YM Hong Y Li J Zhang X. Efficacy, immunogenicity and safety of the HPV-16/18 AS04-adjuvanted vaccine in healthy Chinese women aged 18-25 years: results from a randomized controlled trial. Int J Cancer (2014) 135:2612–22. doi: 10.1002/ijc.28897 PMC4277330 24740596

[36] Zou Z Fairley CK Ong JJ Hocking J Canfell K Ma X. Domestic HPV vaccine price and economic returns for cervical cancer prevention in China: a cost-effectiveness analysis. Lancet Glob Health (2020) 8:e1335–44. doi: 10.1016/s2214-109x(20)30277-1 32971056

[37] Giuliano AR Palefsky JM Goldstone S Moreira ED Penny ME Aranda C. Efficacy of quadrivalent HPV vaccine against HPV infection and disease in males. N Engl J Med (2011) 364:401–11. doi: 10.1056/NEJMoa0909537 PMC3495065 21288094

[38] Soliman M Oredein O Dass CR. Update on safety and efficacy of HPV vaccines: focus on gardasil. Int J Mol Cell Med (2021) 10:101–13. doi: 10.22088/ijmcm.Bums.10.2.101 PMC8496244 34703794

[39] Joura EA Giuliano AR Iversen OE Bouchard C Mao C Mehlsen J. A 9-valent HPV vaccine against infection and intraepithelial neoplasia in women. N Engl J Med (2015) 372:711–23. doi: 10.1056/NEJMoa1405044 25693011

[40] Qbaobei. Price list of domestic HPV vaccine (2021). Available at: http://www.qbaobei.com/jiankang/1441055.html.

[41] WUHAN.com. Hpv bivalent, quadrivalent and nine-valent vaccines price list (2021). Available at: https://www.wuhan.com/life/80158.html.

[42] WHO. WHO guide for standardization of economic evaluations of immunization programmes (2019). Available at: https://apps.who.int/iris/bitstream/handle/10665/329389/WHO-IVB-19.10-eng.pdf.

[43] Ma X Wang Q Ong JJ Fairley CK Su S Peng P. Prevalence of human papillomavirus by geographical regions, sexual orientation and HIV status in China: a systematic review and meta-analysis. Sex Transm Infect (2018) 94:434–42. doi: 10.1136/sextrans-2017-053412 29794242

[44] Guo Z Feng A Zhou Y Gao Y Sun Y Chen Y. Geosocial networking mobile applications use and HIV and other sexually transmitted infections among men who have sex with men in southern China: a cross-sectional study. Front Public Health (2023) 11:1063993. doi: 10.3389/fpubh.2023.1063993 36844866 PMC9944389

[45] Network NCC. Anal carcinoma version 1. 2023-January 9, 2023. (2023) (Pennsylvania: NCCN). Available at: https://www.nccn.org/patients.

[46] Winer RL Lin J Querec TD Unger ER Stern JE Rudd JM. Effectiveness of human papillomavirus (HPV) vaccination against penile HPV infection in men who have sex with men and transgender women. J Infect Dis (2022) 225:422–30. doi: 10.1093/infdis/jiab390 PMC8842819 34320185

[47] Cranston RD Carballo-Diéguez A Gundacker H Richardson BA Giguere R Dolezal C. Prevalence and determinants of anal human papillomavirus infection in men who have sex with men and transgender women. Int J STD AIDS (2019) 30:154–62. doi: 10.1177/0956462418797864 PMC6552657 30336747

[48] Lau JT Wang Z Kim JH Lau M Lai CH Mo PK. Acceptability of HPV vaccines and associations with perceptions related to HPV and HPV vaccines among men who have sex with men in Hong Kong. PloS One (2013) 8:e57204. doi: 10.1371/journal.pone.0057204 23451188 PMC3579800

[49] Li X Cao X Li Z Yang Y Li M Feng B. Human papillomavirus awareness and vaccine acceptability among men who have sex with men from mainland China. Sci Rep (2019) 9:8763. doi: 10.1038/s41598-019-45258-0 31217451 PMC6584641

[50] Soe NN Ong JJ Ma X Fairley CK Latt PM Jing J. Should human papillomavirus vaccination target women over age 26, heterosexual men and men who have sex with men? a targeted literature review of cost-effectiveness. Hum Vaccin Immunother (2018) 14:3010–8. doi: 10.1080/21645515.2018.1496878 PMC6343618 30024823

[51] Ye ZH Liu ZZ Cui ST Chu ZX Jiang YJ Xu JJ. High human papillomavirus vaccine acceptability and cost-effectiveness of the Chinese 2-valent vaccine among men who have sex with men: a cross-sectional study in shenyang, China. Front Med (Lausanne) (2021) 8:763564. doi: 10.3389/fmed.2021.763564 34869470 PMC8639684

[52] Glick SN Morris M Foxman B Aral SO Manhart LE Holmes KK. A comparison of sexual behavior patterns among men who have sex with men and heterosexual men and women. J Acquir Immune Defic Syndr (2012) 60:83–90. doi: 10.1097/QAI.0b013e318247925e 22522237 PMC3334840

[53] Bruzzesi E Galli L Poli A Bossolasco S Cernuschi M Spagnuolo V. Prevalence and risk factors of anal HPV infection in MSM living with HIV: identifying the target groups to prioritize for immunization. J Acquir Immune Defic Syndr (2022) 91:226–31. doi: 10.1097/qai.0000000000003057 35973060

[54] Lin A Ong KJ Hobbelen P King E Mesher D Edmunds WJ. Impact and cost-effectiveness of selective human papillomavirus vaccination of men who have sex with men. Clin Infect Dis (2017) 64:580–8. doi: 10.1093/cid/ciw845 PMC5404831 28011615

[55] Zhu K Tian Y Dong X Akinwunmi BO Zhang CJP Huang J. The cost-effectiveness of bivalent, quadrivalent, and nine-valent HPV vaccination in Asia: a systematic review. Arch Gynecol Obstet (2022) 306:173–87. doi: 10.1007/s00404-021-06309-y 35380278

[56] Organization WH. (2022). Available at: https://www.who.int/.

[57] Barnabas RV Brown ER Onono M Bukusi EA Njoroge B Winer RL. Single-dose HPV vaccination efficacy among adolescent girls and young women in Kenya (the KEN SHE study): study protocol for a randomized controlled trial. Trials (2021) 22:661. doi: 10.1186/s13063-021-05608-8 34579786 PMC8475401

[58] Ben Hadj Yahia MB Jouin-Bortolotti A. Dervaux, b. extending the human papillomavirus vaccination programme to include males in high-income countries: a systematic review of the cost-effectiveness studies. Clin Drug Investig (2015) 35:471–85. doi: 10.1007/s40261-015-0308-4 26187455

