# Supplementary material for: Effectiveness and cost-effectiveness of human papillomavirus vaccination strategies among men who have sex with men in China: a modeling study
Source: Front Immunol. 2023 Jun 22;14:1197191. doi: 10.3389/fimmu.2023.1197191 (PMC10324564; doi:10.3389/fimmu.2023.1197191)
Supplement: Supplementary file 1 [file DataSheet_1.docx]

**Appendix**

**Effectiveness and cost-effectiveness of human papillomavirus vaccination strategies among men who have sex with men in China: a modeling study**

Yuwei Li^1#^, Yi-Fan Lin^1#^, Xinsheng Wu^1^, Xinyi Zhou^1^, Tian Tian^1^, Zhihui Guo^1^, Leiwen Fu^1^, Luoyao Yang^1^, Zhen Lu^1^, Song Fan^2^, Yong Lu^3^, Huachun Zou^1*^

^1^School of Public Health (Shenzhen), Sun Yat-sen University, Shenzhen, China

^2^ School of Public Health, Southwest Medical University, Sichuan, China

^3^College of Public Health and Health Professions, Guizhou Medical University, China

**Contents**

[1. Model structure 2](#_Toc135920054)

[2. Model simulation 5](#_Toc135920056)

[3. Parameters 6](#_Toc135920057)

[3.1 Population size 6](#_Toc135920058)

[3.2 HPV incidence 6](#_Toc135920059)

[3.3 Incremental cost-effectiveness ratio 7](#_Toc135920060)

[3.4 Discount rate 7](#_Toc135920061)

[4. Results 8](#_Toc135920062)

[5. References 15](#_Toc135920063)

# 1. Model Structure

S: susceptible;

L: infected with HPV low-risk subtypes;

H: infected with HPV high-risk subtypes;

G: anogenital warts;

A: anal cancer;

D: death from anal cancer;


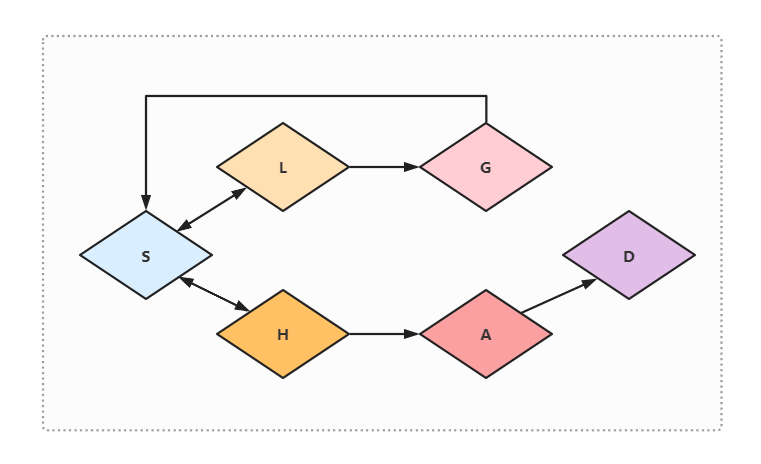


Figure S1. The basic model

1: MSM aged <27 years; 2: MSM aged 27-45 years; 3: MSM aged >45 years.

(A)


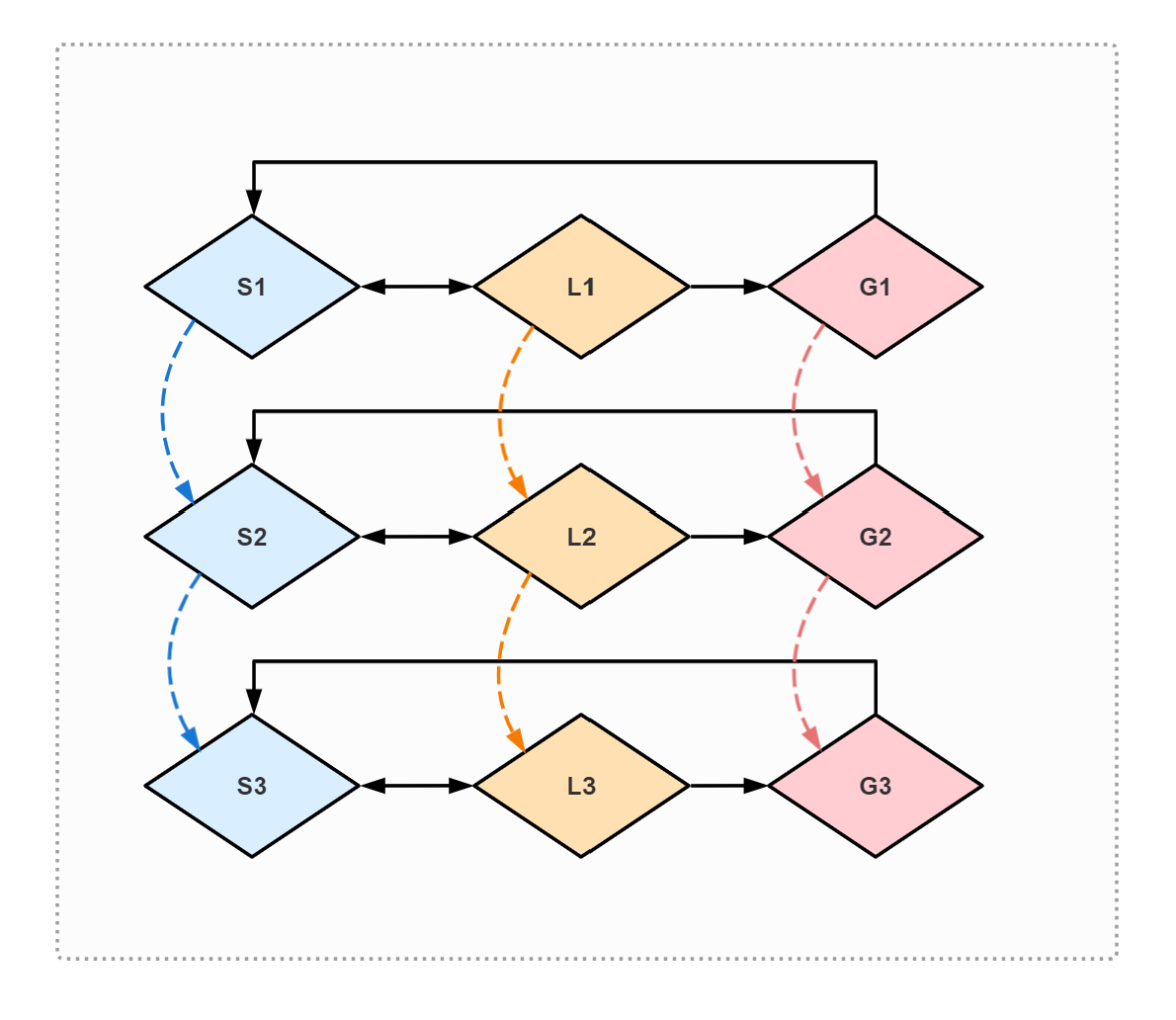


(B)


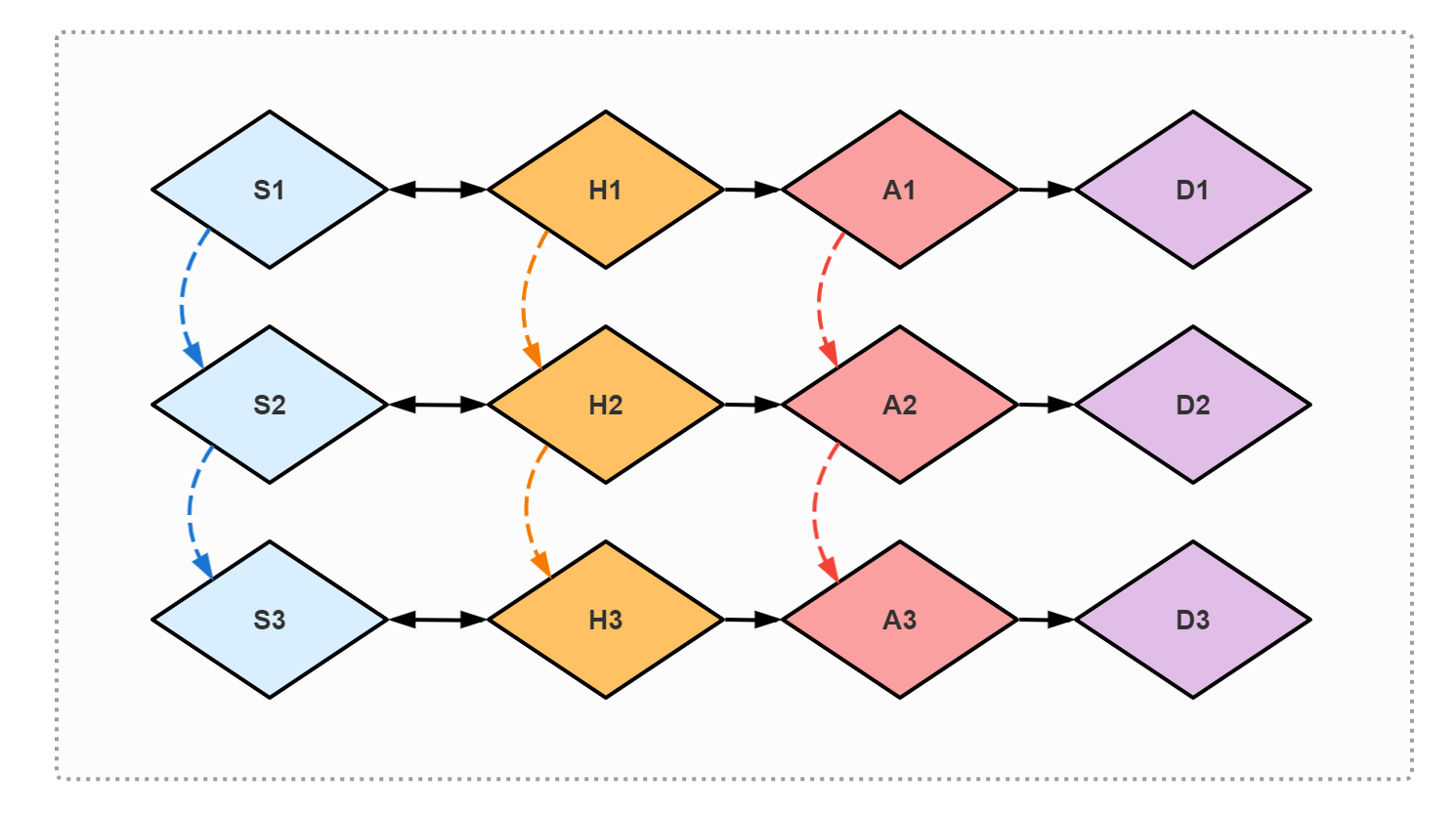


Figure S2. The age-stratified model of the development of low-risk HPV infection (A) /high-risk HPV infection (B).

0: unvaccinated; v: vaccinated;


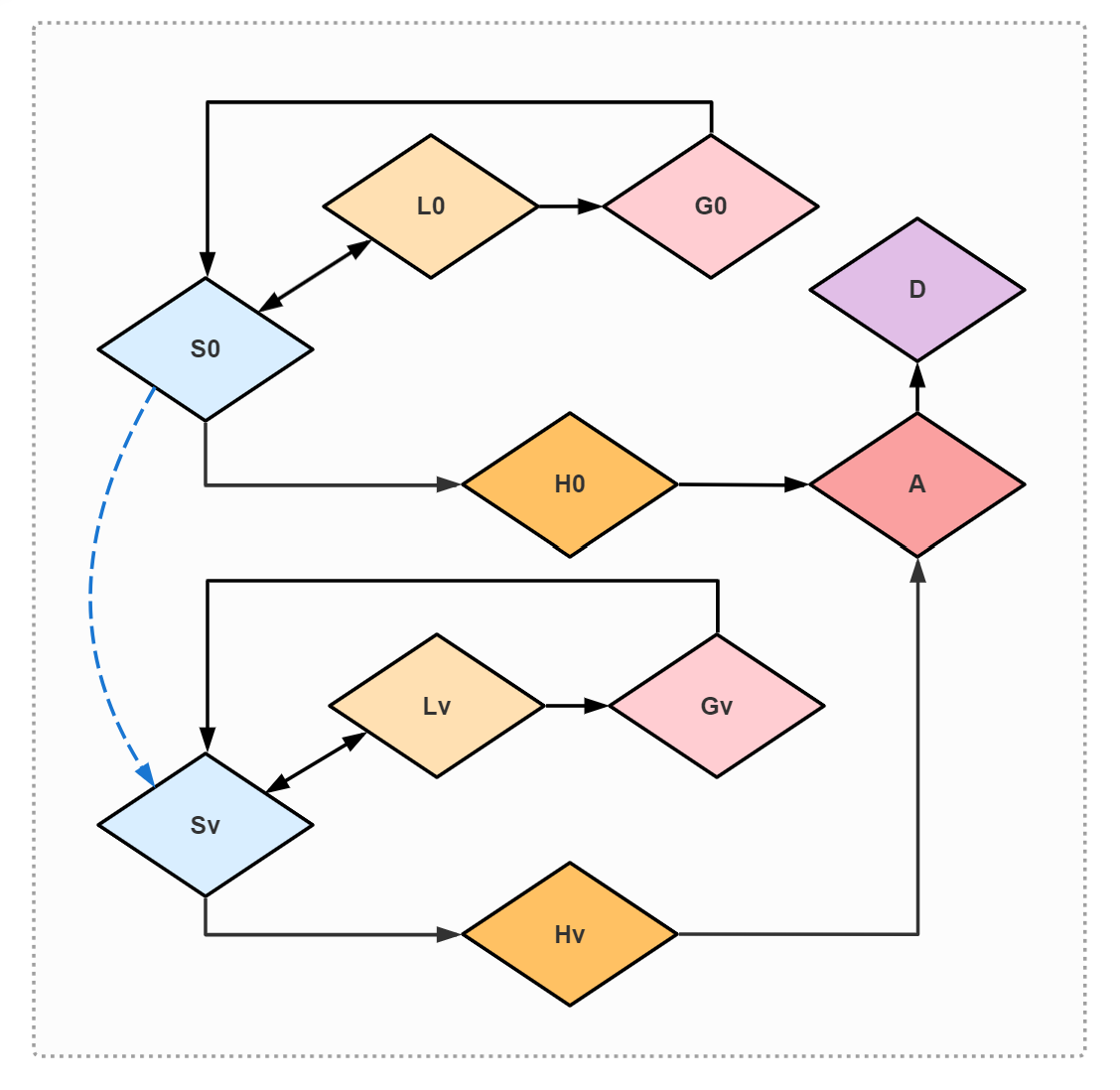


Figure S3. The model with vaccination.

# Model simulation

The Markov matrix used for model simulation was as follows:


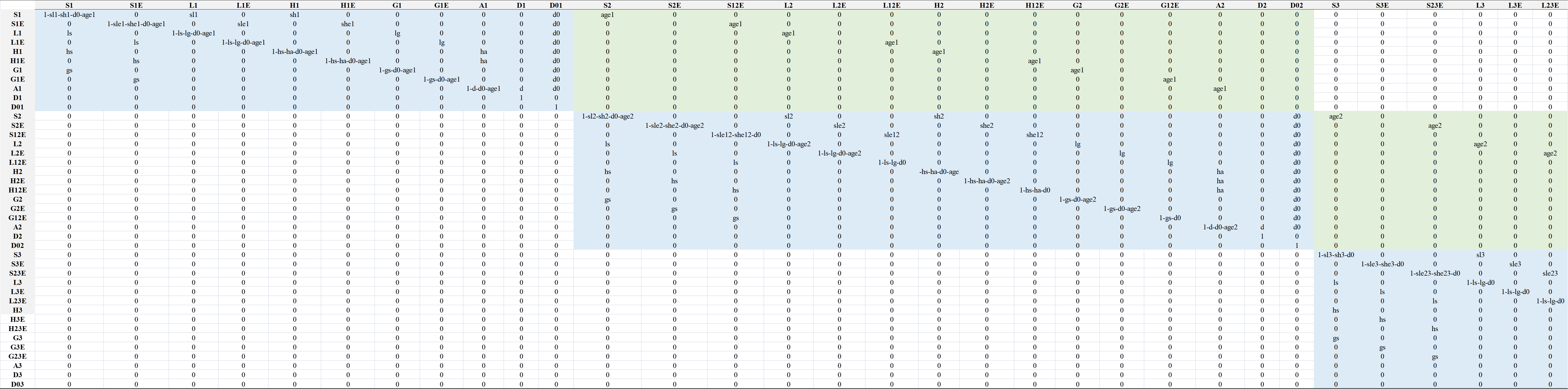


**Figure S4. The age-stratified HPV transmission matrix among MSM**

| Status: S: susceptible; L: low-risk HPV infection; H: high-risk HPV infection; G: anogenital warts; A: anal cancer; D: death from anal cancer; D0: natural death; 1: age group 1, MSM aged <26; 2: age group 2, MSM aged 26-45; 3: age group 3, MSM aged >45; E: vaccinated;  12: those initially in group 1 while transferring to group 2; 23: those initially in group 2 while transferring to group 3. |
| --- |
|  |
|  |
|  |

| **Table S1. Implications for transmission parameters in the matrix** | | |
| --- | --- | --- |
| **Transmission** | **Symbol in Table 1** | **Symbol in matrix** |
| L->G | p_LG_ | lg |
| H->A | p_HA_ | ha |
| L->S | γ_L_ | ls |
| H->S | γ_H_ | hs |
| G->S | γ_G_ | gs |
| A->D | d_A_ | d |
| Group1/2/3->D0 | d_0_ | d0 |
| Group1->Group2 | μ_1_ | age1 |
| Group2->Group3 | μ_2_ | age2 |
| S->SE | β | e |

# 3. Parameters

## 3.1 Population size

The estimation of MSM population size in China:

An online survey in China estimated the nationwide total MSM (n=8,288,536; 95% CI, 8,274,931-8,302,141) using Blued (a social networking MSM app)^1^. A serial cross-sectional study recruited MSM aged ≥18 years through combined offline sampling methods and found that 32.2% of the participants used online social apps, among which 97% used Blued^2^. Therefore, we estimated the number of MSM aged no less than 9 years in China (N) by:

$$N=\frac{n}{32.2\%\times97\%}\times\frac{1}{p} ,$$

where *p* is the coefficient of age stratification, indicating the proportion of MSM aged ≥18 years among MSM aged ≥18 years^3^.

## 3.2 HPV incidence

The incidence rates of different HPV subtypes referred to a 5-year prospective cohort study^4^. The age-stratified HPV incidences were assumed to be 1:2:1 among the <27, 27-45 and >45 age groups, to reflect the higher overall incidences of genital warts and anal cancer among the intermediate male age group (Chesson et al.^5^).

## 3.3 Incremental cost-effectiveness ratio

The calculation of ICER:

The ICER of vaccination strategy A compared with strategy B is:

$$ICER=\frac{(Cost A-Cost B)}{(QALY A-QALY B)} ,$$

where Cost represents the total cost of vaccination plus the cost of treatment for HPV-related diseases, and QALY is the sum of individual quality-adjusted life-years in each status.

## 3.4 Discount rate

The use of discount rate:

A year-end discount rate (0.03) was used to adjust the total cost:

$$Cost=c_{1}+\frac{c_{2}}{(1+0.03)}+\frac{c_{3}}{\left( 1+0.03 \right)^{2}}+\ldots+\frac{c_{10}}{\left( 1+0.03 \right)^{9}} ,$$

where $c_{i}$ denotes the total cost in the ith simulation year.

# 4. Results

**Table S2. The prevented cases and deaths of HPV-related diseases of age-stratified HPV vaccination strategies among MSM in China, compared with no vaccination**

| **Vaccination strategies a** | | | **Prevented anogenital warts (median [IQR])** | **Prevented anal cancers (median [IQR])** | **Prevented deaths from anal cancer (median [IQR])** |
| --- | --- | --- | --- | --- | --- |
| <27 | 27-45 | >45 |  |  |  |
| **One-group-targeted strategies** | | |  |  |  |
| 2 | 0 | 0 | -27,703.30 [-31,392.56, -24,126.55] | 21.95 [19.74, 24.03] | 9.80 [7.54, 11.99] |
| 0 | 2 | 0 | -14,170.52 [-34,266.85, 624.67] | 73.91 [66.77, 80.24] | 31.25 [24.17, 38.18] |
| 0 | 0 | 2 | -2,429.02 [-2,714.53, -2,125.79] | 3.26 [2.94, 3.56] | 1.43 [1.10, 1.75] |
| 4 | 0 | 0 | 102,329.07 [88,727.08, 114,778.45] | 11.34 [9.85, 12.66] | 6.52 [5.10, 7.94] |
| 0 | 4 | 0 | 378,458.92 [314,785.72, 437,167.42] | 33.63 [29.49, 37.57] | 15.61 [12.33, 18.92] |
| 0 | 0 | 4 | 15,292.69 [13,165.93, 17,201.13] | 2.02 [1.80, 2.23] | 1.01 [0.79, 1.23] |
| 9 | 0 | 0 | 81,533.99 [70,405.58, 91,753.41] | 74.24 [66.94, 81.17] | 31.81 [24.49, 38.84] |
| 0 | 9 | 0 | 286,959.37 [232,059.73, 332,853.55] | 231.44 [208.29, 252.58] | 104.80 [81.18, 127.75] |
| 0 | 0 | 9 | 13,479.65 [11,625.96, 15,249.05] | 10.42 [9.39, 11.39] | 4.54 [3.50, 5.54] |
| **Two-group-targeted strategies** | | |  |  |  |
| 2 | 2 | 0 | -41,873.82 [-60,034.07, -27,566.64] | 95.86 [86.37, 104.11] | 41.05 [31.71, 50.18] |
| 2 | 4 | 0 | 350,755.62 [290,315.58, 405,037.38] | 55.58 [49.7, 61.07] | 25.41 [19.97, 30.91] |
| 2 | 9 | 0 | 259,256.07 [207,993.55, 301,422.87] | 253.39 [228.01, 276.43] | 114.60 [88.74, 139.70] |
| 4 | 2 | 0 | 88,158.55 [61,297.79, 107,406.95] | 85.24 [76.63, 93.07] | 37.77 [29.27, 46.11] |
| 4 | 4 | 0 | **480,787.99 [405,437.58, 553,663.48]** | 44.97 [39.71, 49.92] | 22.12 [17.44, 26.87] |
| 4 | 9 | 0 | 389,288.44 [322,937.34, 447,345.88] | 242.78 [217.81, 265.12] | 111.31 [86.30, 135.63] |
| 9 | 2 | 0 | 67,363.47 [42,276.18, 85,364.76] | 148.15 [133.76, 160.98] | 63.06 [48.68, 77.06] |
| 9 | 4 | 0 | 459,992.91 [386,887.89, 530,320.03] | 107.88 [97.02, 117.56] | 47.41 [36.88, 57.70] |
| 9 | 9 | 0 | 368,493.36 [304,160.39, 422,998.40] | **305.69 [275.33, 333.30]** | **136.60 [105.76, 166.55]** |
| 0 | 2 | 2 | -16,599.54 [-36,421.13, -1,760.48] | 77.17 [69.71, 83.83] | 32.68 [25.27, 39.94] |
| 0 | 2 | 4 | 1,122.17 [-19,752.13, 15,461.43] | 75.93 [68.54, 82.50] | 32.26 [24.96, 39.42] |
| 0 | 2 | 9 | -690.87 [-21,537.74, 13,566.73] | 84.33 [76.18, 91.70] | 35.79 [27.67, 43.75] |
| 0 | 4 | 2 | 376,029.90 [312,553.30, 434,360.29] | 36.90 [32.49, 40.97] | 17.04 [13.44, 20.65] |
| 0 | 4 | 4 | 393,751.61 [328,351.66, 453,744.15] | 35.66 [31.35, 39.67] | 16.62 [13.13, 20.14] |
| 0 | 4 | 9 | 391,938.57 [326,874.66, 451,740.96] | 44.05 [39.19, 48.52] | 20.15 [15.86, 24.54] |
| 0 | 9 | 2 | 284,530.36 [229,739.60, 330,343.06] | 234.71 [211.24, 256.19] | 106.23 [82.28, 129.49] |
| 0 | 9 | 4 | 302,252.06 [245,196.13, 348,805.48] | 233.47 [210.04, 254.77] | 105.81 [81.97, 128.98] |
| 0 | 9 | 9 | 300,439.02 [243,736.75, 346,820.88] | 241.86 [217.72, 263.97] | 109.33 [84.69, 133.28] |
| 2 | 0 | 2 | -30,132.32 [-34,142.78, -26,301.18] | 25.21 [22.68, 27.58] | 11.23 [8.65, 13.74] |
| 2 | 0 | 4 | -12,410.61 [-14,211.49, -10,549.70] | 23.97 [21.51, 26.25] | 10.81 [8.34, 13.22] |
| 2 | 0 | 9 | -14,223.65 [-16,285.24, -12,065.37] | 32.37 [29.12, 35.37] | 14.34 [11.04, 17.54] |
| 4 | 0 | 2 | 99,900.05 [86,669.63, 112,098.09] | 14.60 [12.81, 16.23] | 7.95 [6.18, 9.68] |
| 4 | 0 | 4 | 117,621.76 [101,959.41, 132,025.75] | 13.36 [11.63, 14.90] | 7.53 [5.89, 9.18] |
| 4 | 0 | 9 | 115,808.72 [100,397.68, 129,955.85] | 21.75 [19.26, 24.07] | 11.05 [8.58, 13.47] |
| 9 | 0 | 2 | 79,104.97 [68,326.24, 89,093.22] | 77.51 [69.90, 84.72] | 33.24 [25.59, 40.58] |
| 9 | 0 | 4 | 96,826.68 [83,636.47, 108,872.22] | 76.27 [68.70, 83.42] | 32.82 [25.28, 40.07] |
| 9 | 0 | 9 | 95,013.64 [82,008.17, 106,898.11] | 84.66 [76.35, 92.56] | 36.35 [27.99, 44.38] |
| **Full-arrangement strategies** | | |  |  |  |
| 2 | 2 | 2 | -44,302.84 [-62,199.45, -29,563.03] | 99.12 [89.24, 107.67] | 42.48 [32.82, 51.92] |
| 2 | 2 | 4 | -26,581.13 [-45,082.58, -12,309.53] | 97.88 [88.15, 106.39] | 42.06 [32.50, 51.41] |
| 2 | 2 | 9 | -28,394.17 [-46,846.17, -14,365.58] | 106.27 [95.71, 115.43] | 45.59 [35.23, 55.72] |
| 2 | 4 | 2 | 348,326.60 [288,165.14, 402,413.82] | 58.84 [52.67, 64.64] | 26.84 [21.07, 32.65] |
| 2 | 4 | 4 | 366,048.31 [304,014.09, 422,998.20] | 57.60 [51.52, 63.34] | 26.42 [20.76, 32.14] |
| 2 | 4 | 9 | 364,235.27 [302,228.03, 420,923.69] | 66.00 [58.99, 72.27] | 29.95 [23.50, 36.48] |
| 2 | 9 | 2 | 256,827.06 [205,771.87, 298,885.27] | 256.65 [230.96, 279.98] | 116.03 [89.85, 141.45] |
| 2 | 9 | 4 | 274,548.76 [222,142.02, 318,894.87] | 255.41 [229.79, 278.62] | 115.61 [89.53, 140.94] |
| 2 | 9 | 9 | 272,735.72 [220,455.16, 316,569.49] | **263.81 [237.32, 287.77]** | **119.13 [92.26, 145.22]** |
| 4 | 2 | 2 | 85,729.53 [59,165.68, 104,729.51] | 88.51 [79.62, 96.56] | 39.20 [30.37, 47.86] |
| 4 | 2 | 4 | 103,451.24 [75,111.11, 124,771.18] | 87.27 [78.39, 95.26] | 38.78 [30.06, 47.34] |
| 4 | 2 | 9 | 101,638.20 [73,461.61, 122,524.59] | 95.66 [86.05, 104.41] | 42.31 [32.77, 51.66] |
| 4 | 4 | 2 | 478,358.97 [403,372.27, 551,026.75] | 48.23 [42.76, 53.38] | 23.55 [18.58, 28.65] |
| 4 | 4 | 4 | **496,080.68 [418,945.15, 570,872.88]** | 46.99 [41.54, 52.14] | 23.14 [18.23, 28.11] |
| 4 | 4 | 9 | **494,267.64 [417,365.36, 569,060.08]** | 55.39 [49.26, 61.09] | 26.66 [21.03, 32.47] |
| 4 | 9 | 2 | 386,859.43 [320,847.64, 444,902.25] | 246.04 [220.79, 268.68] | 112.74 [87.40, 137.37] |
| 4 | 9 | 4 | 404,581.13 [336,680.94, 465,342.15] | 244.80 [219.64, 267.36] | 112.32 [87.09, 136.87] |
| 4 | 9 | 9 | 402,768.09 [335,003.81, 462,948.72] | 253.20 [227.26, 276.56] | 115.85 [89.80, 141.17] |
| 9 | 2 | 2 | 64,934.45 [40,225.58, 82,673.54] | 151.41 [136.69, 164.54] | 64.49 [49.78, 78.81] |
| 9 | 2 | 4 | 82,656.16 [56,621.78, 102,018.00] | 150.18 [135.55, 163.18] | 64.07 [49.46, 78.29] |
| 9 | 2 | 9 | 80,843.12 [54,977.98, 100,020.83] | 158.57 [143.18, 172.21] | 67.60 [52.17, 82.61] |
| 9 | 4 | 2 | 457,563.89 [384,705.03, 527,640.30] | 111.14 [100.00, 121.04] | 48.84 [37.98, 59.43] |
| 9 | 4 | 4 | **475,285.60 [400,409.88, 548,298.84]** | 109.90 [98.82, 119.73] | 48.43 [37.67, 58.94] |
| 9 | 4 | 9 | **473,472.56 [398,622.30, 546,250.49]** | 118.30 [106.41, 128.79] | 51.95 [40.36, 63.20] |
| 9 | 9 | 2 | 366,064.35 [301,831.89, 420,016.23] | **308.95 [278.24, 336.85]** | **138.03 [106.86, 168.28]** |
| 9 | 9 | 4 | 383,786.05 [317,676.35, 441,279.65] | **307.71 [277.11, 335.56]** | **137.62 [106.55, 167.80]** |
| 9 | 9 | 9 | 381,973.01 [316,108.32, 439,370.04] | **316.11 [284.65, 344.70]** | **141.14 [109.26, 172.08]** |
| HPV, human papillomavirus; MSM, men who have sex with men; IQR: inter-quartile range. A statistically significant increase in the number of anogenital warts is indicated if the upper band of IQR of prevented cases is less than 0; no positive or negative effect of vaccination strategies is suggested if the IQR covers 0. a Vaccination strategies: "<27", "27-45", and ">45" represent the three MSM groups stratified by age (MSM aged <27, 27-45, and >45 years, respectively). Number "0" represents no vaccine. Numbers "2", "4", and "9" represent the bivalent vaccine, quadrivalent vaccine, and nine-valent vaccine, respectively. Each row is an alternative allocation strategy of the three vaccine categories. For example, the line filled with "2", "4", and "2" means allocating bivalent vaccine to MSM aged <27 and >45 years, and quadrivalent vaccine to those aged 27-45 years. | | | | | |
|  |  |  |  |  |  |
|  |  |  |  |  |  |
|  |  |  |  |  |  |
|  |  |  |  |  |  |
|  |  |  |  |  |  |

**Table S3. The gained QALY, increased cost, and ICER of age-stratified HPV vaccination strategies among MSM in China, compared with no vaccination**

| **Vaccination strategies a** | | | **QALY gained (median [IQR])** | **Total cost increased  (million USD, median [IQR])** | **ICER (1000 USD/QALY, median [IQR])** |
| --- | --- | --- | --- | --- | --- |
| <27 | 27-45 | >45 |  |  |  |
| **One-group-targeted strategies** | | |  |  |  |
| 2 | 0 | 0 | -2,476.66 [-2,807.39, -2,155.22] | 254.10 [247.54, 260.67] | / |
| 0 | 2 | 0 | -1,221.08 [-3,019.63, 107.01] | 619.21 [608.05, 630.35] | / |
| 0 | 0 | 2 | -216.16 [-241.83, -189.01] | 36.98 [36.33, 37.63] | / |
| 4 | 0 | 0 | 9,219.62 [7,995.87, 10,339.78] | 439.31 [409.88, 468.87] | 47.65 [44.46, 50.86] |
| 0 | 4 | 0 | 34,087.30 [28,359.07, 39,367.77] | 1,018.22 [920.14, 1,116.75] | 29.87 [26.99, 32.76] |
| 0 | 0 | 4 | 1,377.98 [1,186.62, 1,549.74] | 66.14 [61.77, 70.53] | 48.00 [44.82, 51.18] |
| 9 | 0 | 0 | 7,393.03 [6,391.48, 8,307.74] | 782.27 [757.85, 806.80] | 105.81 [102.51, 109.13] |
| 0 | 9 | 0 | 26,003.11 [21,054.71, 30,169.43] | 1,924.10 [1,851.26, 1,997.25] | 74.00 [71.19, 76.81] |
| 0 | 0 | 9 | 1,220.96 [1,054.77, 1,380.55] | 116.64 [112.74, 120.57] | 95.53 [92.33, 98.75] |
| **Two-group-targeted strategies** | | |  |  |  |
| 2 | 2 | 0 | -3,697.74 [-5,329.05, -2,401.87] | 873.32 [855.60, 891.05] | / |
| 2 | 4 | 0 | 31,610.64 [26,176.31, 36,484.84] | 1,272.33 [1,180.82, 1,364.24] | 40.25 [37.36, 43.16] |
| 2 | 9 | 0 | 23,526.45 [18,916.81, 27,320.99] | 2,178.21 [2,111.95, 2,244.73] | 92.59 [89.77, 95.41] |
| 4 | 2 | 0 | 7,998.54 [5,585.02, 9,736.05] | 1,058.52 [1,040.27, 1,076.83] | 132.34 [130.06, 134.63] |
| 4 | 4 | 0 | 43,306.92 [36,523.99, 49,860.28] | 1,457.53 [1,330.01, 1,585.62] | 33.66 [30.71, 36.61] |
| 4 | 9 | 0 | 35,222.73 [29,240.62, 40,466.75] | 2,363.41 [2,261.14, 2,466.12] | 67.10 [64.20, 70.01] |
| 9 | 2 | 0 | 6,171.95 [3,926.34, 7,794.10] | 1,401.49 [1,388.12, 1,414.83] | 227.07 [224.91, 229.24] |
| 9 | 4 | 0 | 41,480.33 [34,913.76, 47,812.63] | 1,800.49 [1,677.98, 1,923.55] | 43.41 [40.45, 46.37] |
| 9 | 9 | 0 | 33,396.14 [27,590.13, 38,295.91] | 2,706.37 [2,609.11, 2,804.05] | 81.04 [78.13, 83.96] |
| 0 | 2 | 2 | -1,437.24 [-3,221.40, -97.71] | 656.19 [644.38, 667.99] | / |
| 0 | 2 | 4 | 156.90 [-1,722.66, 1,444.78] | 685.35 [678.54, 692.11] | 4,367.99 [4,324.59, 4,411.02] |
| 0 | 2 | 9 | -0.12 [-1,874.88, 1,286.57] | 735.86 [728.59, 743.07] | / |
| 0 | 4 | 2 | 33,871.13 [28,160.24, 39,115.12] | 1,055.20 [957.77, 1,153.07] | 31.15 [28.28, 34.04] |
| 0 | 4 | 4 | 35,465.28 [29,577.88, 40,863.80] | 1,084.36 [981.90, 1,187.28] | 30.58 [27.69, 33.48] |
| 0 | 4 | 9 | 35,308.25 [29,452.22, 40,683.88] | 1,134.87 [1,032.87, 1,237.32] | 32.14 [29.25, 35.04] |
| 0 | 9 | 2 | 25,786.94 [20,876.56, 29,919.00] | 1,961.08 [1,888.90, 2,033.57] | 76.05 [73.25, 78.86] |
| 0 | 9 | 4 | 27,381.09 [22,249.35, 31,571.11] | 1,990.24 [1,913.03, 2,067.78] | 72.69 [69.87, 75.52] |
| 0 | 9 | 9 | 27,224.06 [22,112.47, 31,391.18] | 2,040.75 [1,964.00, 2,117.82] | 74.96 [72.14, 77.79] |
| 2 | 0 | 2 | -2,692.83 [-3,055.41, -2,348.21] | 291.09 [283.87, 298.31] | / |
| 2 | 0 | 4 | -1,098.68 [-1,261.05, -931.09] | 320.24 [318.04, 322.43] | / |
| 2 | 0 | 9 | -1,255.70 [-1,439.59, -1,062.61] | 370.75 [368.08, 373.39] | / |
| 4 | 0 | 2 | 9,003.46 [7,811.38, 10,100.80] | 476.29 [447.51, 505.19] | 52.90 [49.70, 56.11] |
| 4 | 0 | 4 | 10,597.60 [9,187.80, 11,895.21] | 505.45 [471.64, 539.40] | 47.69 [44.50, 50.90] |
| 4 | 0 | 9 | 10,440.58 [9,055.37, 11,712.23] | 555.95 [522.61, 589.44] | 53.25 [50.06, 56.46] |
| 9 | 0 | 2 | 7,176.87 [6,208.70, 8,071.98] | 819.25 [795.48, 843.12] | 114.15 [110.84, 117.48] |
| 9 | 0 | 4 | 8,771.01 [7,587.24, 9,855.45] | 848.41 [819.61, 877.33] | 96.73 [93.45, 100.03] |
| 9 | 0 | 9 | 8,613.99 [7,449.54, 9,677.91] | 898.91 [870.58, 927.37] | 104.36 [101.07, 107.66] |
| **Three-group-targeted strategies** | | |  |  |  |
| 2 | 2 | 2 | -3,913.90 [-5,523.97, -2,590.51] | 910.30 [891.93, 928.68] | / |
| 2 | 2 | 4 | -2,319.76 [-3,987.22, -1,036.81] | 939.46 [926.08, 952.77] | / |
| 2 | 2 | 9 | -2,476.78 [-4,146.38, -1,211.59] | 989.96 [976.12, 1003.73] | / |
| 2 | 4 | 2 | 31,394.47 [25,980.31, 36,265.47] | 1,309.31 [1,218.46, 1,400.57] | 41.71 [38.81, 44.61] |
| 2 | 4 | 4 | 32,988.62 [27,411.34, 38,117.65] | 1,338.47 [1,242.59, 1,434.77] | 40.57 [37.67, 43.49] |
| 2 | 4 | 9 | 32,831.59 [27,257.21, 37,925.48] | 1,388.97 [1,293.56, 1,484.81] | 42.31 [39.40, 45.23] |
| 2 | 9 | 2 | 23,310.28 [18,720.71, 27,111.30] | 2,215.19 [2,149.59, 2,281.05] | 95.03 [92.22, 97.86] |
| 2 | 9 | 4 | 24,904.43 [20,182.16, 28,891.94] | 2,244.35 [2,173.72, 2,315.26] | 90.12 [87.28, 92.97] |
| 2 | 9 | 9 | 24,747.40 [20,056.01, 28,694.71] | 2,294.85 [2,224.69, 2,365.30] | 92.73 [89.90, 95.58] |
| 4 | 2 | 2 | 7,782.38 [5,396.15, 9,492.64] | 1,095.50 [1,077.89, 1,113.15] | 140.77 [138.50, 143.04] |
| 4 | 2 | 4 | 9,376.53 [6,833.79, 11,297.14] | 1,124.66 [1,102.05, 1,147.36] | 119.94 [117.53, 122.37] |
| 4 | 2 | 9 | 9,219.50 [6,685.08, 11,100.24] | 1,175.17 [1,153.02, 1,197.40] | 127.47 [125.06, 129.88] |
| 4 | 4 | 2 | 43,090.76 [36,340.46, 49,625.08] | 1,494.51 [1,367.65, 1,621.94] | 34.68 [31.74, 37.64] |
| 4 | 4 | 4 | 44,684.90 [37,740.83, 51,416.49] | 1,523.67 [1,391.78, 1,656.15] | 34.10 [31.15, 37.06] |
| 4 | 4 | 9 | 44,527.88 [37,605.44, 51,261.67] | 1,574.17 [1,442.75, 1,706.19] | 35.35 [32.40, 38.32] |
| 4 | 9 | 2 | 35,006.56 [29,048.27, 40,222.39] | 2,400.39 [2,298.78, 2,502.44] | 68.57 [65.67, 71.48] |
| 4 | 9 | 4 | 36,600.71 [30,500.92, 42,044.58] | 2,429.55 [2,322.91, 2,536.65] | 66.38 [63.47, 69.31] |
| 4 | 9 | 9 | 36,443.69 [30,354.44, 41,851.75] | 2,480.05 [2,373.88, 2,586.69] | 68.05 [65.14, 70.98] |
| 9 | 2 | 2 | 5,955.79 [3,734.66, 7,547.41] | 1,438.47 [1,425.74, 1,451.16] | 241.52 [239.39, 243.66] |
| 9 | 2 | 4 | 7,549.94 [5,211.49, 9,295.70] | 1,467.62 [1,449.89, 1,485.29] | 194.39 [192.04, 196.73] |
| 9 | 2 | 9 | 7,392.91 [5,074.93, 9,121.07] | 1,518.13 [1,500.84, 1,535.34] | 205.35 [203.01, 207.68] |
| 9 | 4 | 2 | 41,264.17 [34,716.50, 47,577.70] | 1,837.47 [1,715.62, 1,959.87] | 44.53 [41.58, 47.50] |
| 9 | 4 | 4 | 42,858.31 [36,120.90, 49,426.27] | 1,866.63 [1,739.75, 1,994.08] | 43.55 [40.59, 46.53] |
| 9 | 4 | 9 | 42,701.29 [35,969.60, 49,248.69] | 1,917.14 [1,790.72, 2,044.12] | 44.90 [41.94, 47.87] |
| 9 | 9 | 2 | 33,179.97 [27,414.00, 38,034.81] | 2,743.35 [2,646.75, 2,840.37] | 82.68 [79.77, 85.60] |
| 9 | 9 | 4 | 34,774.12 [28,816.47, 39,941.29] | 2,772.51 [2,670.88, 2,874.58] | 79.73 [76.81, 82.66] |
| 9 | 9 | 9 | 34,617.10 [28,669.36, 39,768.69] | 2,823.02 [2,721.85, 2,924.62] | 81.55 [78.63, 84.48] |
| HPV, human papillomavirus; MSM, men who have sex with men; IQR: inter-quartile range; QALY, quality-adjusted life years; ICER, incremental cost-effectiveness ratio. The ICER values in red are less than three times the per capita GDP (gross domestic product) in China. "/" indicates that the calculation of ICER is meaningless, as no gained QALY is detected. a Vaccination strategies: "<27", "27-45", and ">45" represent the three MSM groups stratified by age (MSM aged <27, 27-45, and >45 years, respectively). Number "0" represents no vaccine. Numbers "2", "4", and "9" represent the bivalent vaccine, quadrivalent vaccine, and nine-valent vaccine, respectively. Each row is an alternative allocation strategy of the three vaccine categories. For example, the line filled with "2", "4", and "2" means allocating bivalent vaccine to MSM aged <27 and >45 years, and quadrivalent vaccine to those aged 27-45 years. | | | | | |

# 5. References

1 Hu, M., Xu, C.,Wang, J. Spatiotemporal Analysis of Men Who Have Sex With Men in Mainland China: Social App Capture-Recapture Method. *JMIR Mhealth Uhealth* **8**, e14800, doi:10.2196/14800 (2020).

2 Wei, L., Chen, L., Zhang, H. *et al.* Relationship between gay app use and HIV testing among men who have sex with men in Shenzhen, China: a serial cross-sectional study. *BMJ Open* **9**, e028933, doi:10.1136/bmjopen-2019-028933 (2019).

3 China, N. B. o. S. o. *Statistical Year Book (2022)*, <<http://www.stats.gov.cn/>> (2022).

4 Zhang, Z., Ling, X., Liu, L. *et al.* Natural History of Anal Papillomavirus Infection in HIV-Negative Men Who Have Sex With Men Based on a Markov Model: A 5-Year Prospective Cohort Study. *Front Public Health* **10**, 891991, doi:10.3389/fpubh.2022.891991 (2022).

5 Chesson, H. W., Markowitz, L. E., Hariri, S. *et al.* The impact and cost-effectiveness of nonavalent HPV vaccination in the United States: Estimates from a simplified transmission model. *Hum Vaccin Immunother* **12**, 1363-1372, doi:10.1080/21645515.2016.1140288 (2016).
